# Landauer‐QFLPS Model for Mixed Schottky‐Ohmic Contact Two‐Dimensional Transistors

**DOI:** 10.1002/advs.202303734

**Published:** 2023-10-09

**Authors:** Zhao‐Yi Yan, Zhan Hou, Kan‐Hao Xue, He Tian, Tian Lu, Junying Xue, Fan Wu, Ruiting Zhao, Minghao Shao, Jianlan Yan, Anzhi Yan, Zhenze Wang, Penghui Shen, Mingyue Zhao, Xiangshui Miao, Zhaoyang Lin, Houfang Liu, Yi Yang, Tian‐Ling Ren

**Affiliations:** ^1^ School of Integrated Circuits Tsinghua University Beijing 100084 China; ^2^ Beijing National Research Center for Information Science and Technology (BNRist) Tsinghua University Beijing 100084 China; ^3^ School of Integrated Circuits Huazhong University of Science and Technology Wuhan 430074 China; ^4^ Hubei Yangtze Memory Laboratories Wuhan 430205 China; ^5^ Department of Chemistry Tsinghua University Beijing 100084 China

**Keywords:** ambipolar transport, contact transports, electronic design automation, field‐effect transistors, quasi‐Fermi levels, Schottky barriers, two‐dimensional materials

## Abstract

Two‐dimensional material‐based field‐effect transistors (2DM‐FETs) are playing a revolutionary role in electronic devices. However, before electronic design automation (EDA) for 2DM‐FETs can be achieved, it remains necessary to determine how to incorporate contact transports into model. Reported methods compromise between physical intelligibility and model compactness due to the heterojunction nature. To address this, quasi‐Fermi‐level phase space theory (QFLPS) is generalized to incorporate contact transports using the Landauer formula. It turns out that the Landauer‐QFLPS model effectively overcomes the issue of concern. The proposed new formula can describe 2DM‐FETs with Schottky or Ohmic contacts with superior accuracy and efficiency over previous methods, especially when describing non‐monotonic drain conductance characteristics. A three‐bit threshold inverter quantizer (TIQ) circuit is fabricated using ambipolar black phosphorus and it is demonstrated that the model accurately predicts circuit performance. The model could be very effective and valuable in the development of 2DM‐FET‐based integrated circuits.

## Graphical abstract

A key technique named as Landauer‐QFLPS method in developing EDA tools for two‐dimensional (2D) electronics is proposed. It is shown to be able to capture the contact effect caused by the Schottky barriers formed at the source and drain in 2D‐FETs, which has yet to be canonically governed by a 2D‐FET computing model that fully utilizes its 2D characteristics.

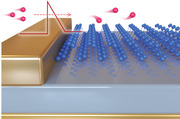

## 1 Introduction

Two‐dimensional material‐based field‐effect transistors (2DM‐FETs) have attracted significant attention for their potential to further increase the number of transistors that can be added to an integrated circuit.^[^ ^1^, ^2^ ^]^ Their atomic thickness and dangling‐bond‐free interface with gate oxide enable high tunability when applied in FET devices.^[^ ^3^ ^]^ For example, ambipolar 2DM‐FETs that can transport electrons and holes simultaneously are extensively reported; these use a variety of channel materials such as graphene (Gr),^[^ ^4^ ^]^ black phosphorus (BP),^[^ ^5^ ^]^ tungsten diselenide (WSe_2_),^[^ ^6^ ^]^ and molybdenum ditelluride (MoTe_2_).^[^ ^7^ ^]^ This has opened a new way of fabricating highly efficient computational components, which could benefit broad applications, including signal processing,^[^ ^8^, ^9^, ^10^, ^11^, ^12^ ^]^ communication,^[^ ^13^ ^]^ hardware‐security,^[^ ^14^ ^]^ 2DM‐based memory,^[^ ^15^ ^]^ and in‐memory computing.^[^ ^16^, ^17^ ^]^ In terms of system‐level applications, electronic design automation (EDA) tools for 2DM‐FETs are in high demand, where an analytical model seizing the physical uniqueness of 2DMs is the crown jewels. A great example is the well‐known Pao‐Sah equation,^[^ ^18^, ^19^, ^20^ ^]^ upon which the SPICE (Simulation Program with Integrated Circuit Emphasis) models are based.^[^ ^21^ ^]^ The unparalleled simulation speed of analytical models somehow lies at the heart of such success.

Although the Pao‐Sah equation simply assumes ideal Ohmic contacts, 2DM/metal contacts tend to involve the Schottky barrier effect; thus, another analytical model is needed for practical application of 2DM‐FETs. Output curves for fabricated 2DM‐FETs tend to show remarkable nonlinearity due to significant Schottky barriers.^[^ ^22^, ^23^, ^24^, ^25^, ^26^, ^27^, ^28^, ^29^, ^30^, ^31^, ^32^ ^]^ To get around this, recent studies have introduced Bi, Sn, and Y elements to achieve Ohmic contacts,^[^ ^33^, ^34^, ^35^ ^]^ but the systems have had process‐compatibility issues. For example, Bi can suppress the generation of metal‐induced gap states (MIGS) due to its semi‐metallic band dispersion characteristics, resulting in Ohmic contacts.^[^ ^35^ ^]^ However, both Bi and Sn are low‐melting‐point metals that are incompatible with back‐end‐of‐line (BEOL) processing, hindering the practical implementation of this technology.^[^ ^36^ ^]^ In conventional contact processes with large‐scale production potential, Schottky characteristics are still common. Hence, the drain current of a 2DM‐FET would be overestimated if the contacts were simply treated as Ohmic.^[^ ^2^ ^]^

One way to address this is to treat the contacts as the rate‐determining step of the carrier transport process to determine drain currents. This can be done using certain approaches that employ the Landauer formula,^[^ ^37^, ^38^, ^39^, ^40^ ^]^ which completely ignores the drift‐diffusion process in the channel. The problem there, however, lies in the unknown partitions of drain‐source bias *V*_DS_ dropping across the contacts. It is only when the transport is strictly ballistic that such a method works, as it requires the channel length to be smaller than the mean‐free‐path of carriers. However, most practical devices do not meet this criterion. Therefore, another method is needed.

Recently, the quasi‐Fermi level phase space (QFLPS) theory was proposed,^[^ ^41^ ^]^ which permits a much more efficient description of channel electrostatic modulation in a 2DM‐FET. A brief introduction of QFLPS is given in the Experimental Section. In this work, QFLPS theory is generalized to incorporate contact transports. Normally, this would lead to labored computational loads, beyond the ability of EDA software to process efficiently. However, we developed an analytical formula that involves contact effects by relying on the specific density states of 2DMs. The unique 2D electronic structure helps to eliminate the contact dilemma currently intrinsic to 2DM‐FETs.

## 2 Landauer‐QFLPS Modeling

**Figure 1:**
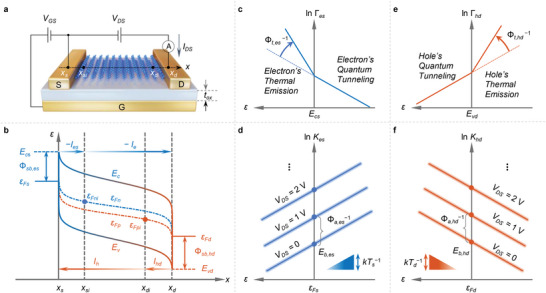
Schematic diagrams of the Landauer‐QFLPS model. a) Structure of 2DM‐FET device and electrical testing schematic; b) Energy band diagram of current transport mechanism; c) Electron transmission energy spectrum of source electrons; d) Collective kinetic energy spectrum of source electrons; e) Transmission energy spectrum of drain holes; f) Collective kinetic energy spectrum of drain holes.

Our approach is named the Landauer‐QFLPS model, constructed as follows. The channel region is divided into three parts: the source‐contact region [*x*_s_,*x*_si_], the intrinsic channel [*x*_si_,*x*_di_], and the drain‐contact region [*x*_di_,*x*_d_]. With the gate‐source voltage *V*_GS_ and drain‐source voltage *V*_DS_ (assuming *V*_DS_ > 0 for clarity) applied, the current *I*_DS_ flows from the drain to the source (as shown in **Figure** **1**a) and includes both electron and hole components, i.e., *I*_DS_ = *I*_e_  + *I*_h_. It is assumed that the Schottky barrier at the source blocks the injection of electrons into the channel, but finite electron flow is present due to either thermal or tunneling effects, leading to the electron‐injection current *I*_es_ on the [*x*_s_,*x*_si_] interval. At the same time, electron‐QFL ε_Fn_ drops from ε_Fs_ at *x*_s_ to ε_Fni_ at *x*_si_ (the blue dotted line in Figure 1b). Similarly, holes overcoming the Schottky barrier at the drain generates a hole‐injection current *I*_hd_ at the [*x*_di_,*x*_d_] interval and the hole‐QFL ε_Fp_ rises from ε_Fd_ at *x*_d_ to ε_Fpi_ at *x*_di_ (the red dotted line in Figure 1b). The internal QFLs, ε_Fni_ and ε_Fpi_, are implicitly determined through the conservation laws of electron and hole currents, respectively, i.e., *I*_es_ = *I*_e_  and *I*_hd_ = *I*_h_ . Once ε_Fni_ and ε_Fpi_ are determined, the conservation quantities *I*_e_ and *I*_h_ (or *I*_es_ and *I*_hd_) can be obtained, yielding *I*_DS_. However, solving the internal QFLs is cumbersome. A central message of this work is that *I*_DS_ can be determined without explicitly finding ε_Fni_ and ε_Fpi_. Here, the intrinsic channel currents *I*_e_ and *I*_h_ are already formulated by the QFLPS model, i.e., the integrals of carrier densities with their QFLs’ paths. Therefore, the key proposal is the development of QFLPS‐model‐like forms for the contact currents *I*_es_ and *I*_hd_, which is achieved through some mathematical derivations.

For simplicity, we focus on the case for electrons, but the derivation for holes is similar. As the injection of electrons from the source to the channel occurs in an atomically thin space,^[^ ^42^ ^]^ the relation between the amount of QFL lowering and the resulting current *I*_es_ should be described by the Landauer formula^[^ ^37^]

(1) $$I_{\mathrm{es}}=W\frac{q}{\pi \hbar }\int_{-\infty }^{+\infty }\Gamma_{\mathrm{es}}\left(\varepsilon \right)M_{\mathrm{es}}\left(\varepsilon \right)\left[f\left(\varepsilon ,\varepsilon_{\mathrm{Fs}}\right)-f\left(\varepsilon ,\varepsilon_{\mathrm{Fni}}\right)\right]d\varepsilon$$

where *W* is the channel width, *q* is the elementary charge, ℏ is the reduced Planck constant, Γ_es_(ε) is the transmission energy spectrum of source electrons, and *M*_es_(ε) is the source electron density‐of‐mode (DOM) energy spectrum. Function *f* (ε, ε_F_) = (1 + exp (ε − ε_*F*_)/*kT*)^−1^  represents the Fermi‐Dirac distribution, where *k* is the Boltzmann constant, and *T* is absolute temperature.

The transmission energy spectrum Γ_es_(ε) is modeled considering the following fact: for electron energy higher than the source‐contact barrier (ε > *E*_cs_, where *E*_cs_ represents the conduction band edge *E*_c_ at *x*_s_ and the Schottky barrier for source electrons $\Phi_{\mathrm{sb},\mathrm{es}}≔E_{\mathrm{cs}}-\varepsilon_{\mathrm{Fs}}$, as shown in Figure 1b), thermal emission dominates; while for electron energy lower than the barrier (ε < *E*_cs_), quantum tunneling dominates. This physical picture (Figure 1c) can be summarized through the following equation

(2) $$\Gamma_{\mathrm{es}}\;\left(\varepsilon \right)=\exp\left(-2\gamma +\Lambda \left(\varepsilon -E_{\mathrm{cs}}\right)/\Phi_{t,\mathrm{es}}\right)$$

where $\gamma ={\left(2\hbar^{2}q\rho_{s}/m_{e}^{*}m_{0}\varepsilon_{s}\right)}^{-1/2}\;\left(E_{\mathrm{cs}}-\varepsilon \right)$ originates from Wentzel–Kramers–Brillouin (WKB) approximation (Note S1, Supporting Information). Here, *m*_0_, $m_{e}^{*}$, ρ_s_, and ε_s_ represent the electron rest mass, source electron relative effective mass, local effective charge density, and dielectric constant, respectively. Λ(·) is the ramp function, and Φ_t,es_ represents the thermal‐emission energy barrier for the source electrons. The thermal emission mechanism signified by the Λ‐term enters the transmission energy spectrum as an augmentation factor.

The DOM function *M*_es_(ε) is given through the formula^[^ ^38^ ^]^

(3) $$M_{\mathrm{es}}\;\left(\varepsilon \right)=\frac{g_{v}}{\pi h}\;\sqrt{2m_{\mathrm{es}}^{*}m_{0}K_{\mathrm{es}}\left(\varepsilon \right)}$$

where *g*_v_ is the valley degeneracy, $m_{\mathrm{es}}^{*}$ is the relative effective mass of source electrons, and *K*_es_(ε) is the collective energy spectrum of source electrons,^[^ ^38^, ^43^ ^]^ taking into account the energy‐level occupancy described by the Maxwell–Boltzmann distribution in thermal equilibrium. However, what is of particular interest is the situation where the system is driven away from thermal equilibrium by applied *V*_DS_. Therefore, the *K*_es_(ε) spectrum should be modified accordingly to lift the collective momentum to a higher level, as shown in Figure 1d. With this acceleration effect, *K*_es_(ε) is written as

(4) $$K_{\mathrm{es}}\;\left(\varepsilon \right)=E_{b,\mathrm{es}}\;\exp\left(\frac{qV_{\mathrm{DS}}}{\Phi_{a,\mathrm{es}}}\right)\exp\left(-\frac{\varepsilon -\varepsilon_{\mathrm{Fs}}}{kT_{s}}\right)$$

where *E*_b,es_ represents the baseline kinetic energy at *V*_DS_ =  0, and Φ_a,es_ represents the acceleration barrier for the source electrons, while *T*_s_ is the temperature of the source contact.^[^ ^44^, ^45^ ^]^ Based on Equations (2)−(4) and 2D density‐of‐states (DOS) of the channel carrier density model (cf. Experimental Section), the electron‐injection current *I*_es_ at the source contact defined by Equation (1) can be transformed into a QFL‐integral form, like in the QFLPS approach, as (Note S1, Supporting Information)

(5) $$I_{\mathrm{es}}=\exp\left(-\eta_{\mathrm{es}}+\frac{qV_{\mathrm{DS}}}{\Phi_{a,\mathrm{es}}}\right)\;\frac{W}{L}\int_{\varepsilon_{\mathrm{Fni}}}^{\varepsilon_{\mathrm{Fs}}}\mu_{n}nd\varepsilon_{\mathrm{Fn}}$$

with the source electron contact‐current‐limiting (CCL) index η_es_ defined as

(6) $$\eta_{\mathrm{es}}=\ln\left[\frac{m_{e}^{*}\mu_{n}/q}{L\frac{\sqrt{2m_{\mathrm{es}}^{*}E_{b,\mathrm{es}}}}{8\pi^{2}\Phi_{t,\mathrm{es}}}\exp\left(-\frac{\Phi_{\mathrm{sb},\mathrm{es}}}{2kT_{s}}\right)}\right]$$

where μ_n_ is the electron mobility, *n* is the 2D‐electron density, *L* is the channel length, and the reciprocal of the pre‐exponential factor of Equation (5), i.e., exp (η_es_ − *qV*_DS_/Φ_a,es_), is defined as the barrier attenuation factor (BAF) for source electrons. After electron injection from the source into the channel, the current *I*_es_ is converted into the intrinsic‐channel current *I*_e_, which is described by the QFLPS model,^[^ ^41^ ^]^ i.e., an integral of (*W*/*L*)μ_n_ *n* over the interval [ε_Fd_, ε_Fni_]. Using the electron current conservation condition *I*_e_ = *I*_es_ , the internal QFL ε_Fni_ can be implicitly eliminated from Equation (5), which leads to the electron's Landauer‐QFLPS formula (Note S1, Supporting Information)

(7) $$I_{e}=\frac{1}{1+\exp\left(\eta_{\mathrm{es}}-qV_{\mathrm{DS}}/\Phi_{a,\mathrm{es}}\right)}\;\frac{W}{L}\int_{\varepsilon_{\mathrm{Fd}}}^{\varepsilon_{\mathrm{Fs}}}\mu_{n}nd\varepsilon_{\mathrm{Fn}}$$

Similarly, for holes, the physical picture drawn above needs an inversion in the energy dimension. Take the transmission spectrum Γ_hd_(ε) of holes injected from the drain as an example (Figure 1e). The thermal emission mechanism is activated when the energy is lower than the valence band maximum, while quantum tunneling dominates when the energy is higher than it. The adaptions are similar for *K*_hd_(ε) (Figure 1f). Hence, the Landauer‐QFLPS model formula for holes is derived as (Note S2, Supporting Information)

(8) $$I_{h}=\frac{1}{1+\exp\left(\eta_{\mathrm{hd}}-qV_{\mathrm{DS}}/\Phi_{a,\mathrm{hd}}\right)}\;\frac{W}{L}\int_{\varepsilon_{\mathrm{Fd}}}^{\varepsilon_{\mathrm{Fs}}}\mu_{p}pd\varepsilon_{\mathrm{Fp}}$$

with the drain hole CCL index η_hd_ defined as

(9) $$\eta_{\mathrm{hd}}=\ln\left[\frac{m_{h}^{*}m_{0}\mu_{p}/q}{L\frac{\sqrt{2m_{\mathrm{hd}}^{*}m_{0}E_{b,\mathrm{hd}}}}{8\pi^{2}\Phi_{t,\mathrm{hd}}}\exp\left(-\frac{\Phi_{\mathrm{sb},\mathrm{hd}}}{2kT_{d}}\right)}\right]$$

where μ_p_ is hole mobility, *p* is the 2D hole density, $m_{h}^{*}$ is the relative effective mass of channel holes, Φ_a,hd_ is the acceleration barrier for the drain holes, Φ_t,hd_ is the thermal emission barrier for drain holes, $m_{\mathrm{hd}}^{*}$ is the relative effective mass of drain holes, *E*_b,hd_ is the baseline thermal equilibrium kinetic energy of drain holes, *T*_d_ is the temperature of the drain contact, and Φ_sb,hd_ is the Schottky barrier for drain holes.

In sum, the Landauer‐QFLPS model gives the total current as *I*_DS_ = *I*_e_  + *I*_h_, where *I*_e_ and *I*_h_ are described by Equations (7) and (8), respectively. They differ from the QFLPS model^[^ ^41^ ^]^ in terms of the respective pre‐factors, which are always less than 1 and describe the contact effects at the source and the drain. For simplicity, η_es_ and η_hd_ are assumed to be identical in the model so that a unique η  = η_es_  = η_hd_  is defined to quantify the overall contact effect strength. The technical details for model parameter extraction are presented in the Experimental Section.

## 3 Transistor‐Level Verification

**Figure 2:**
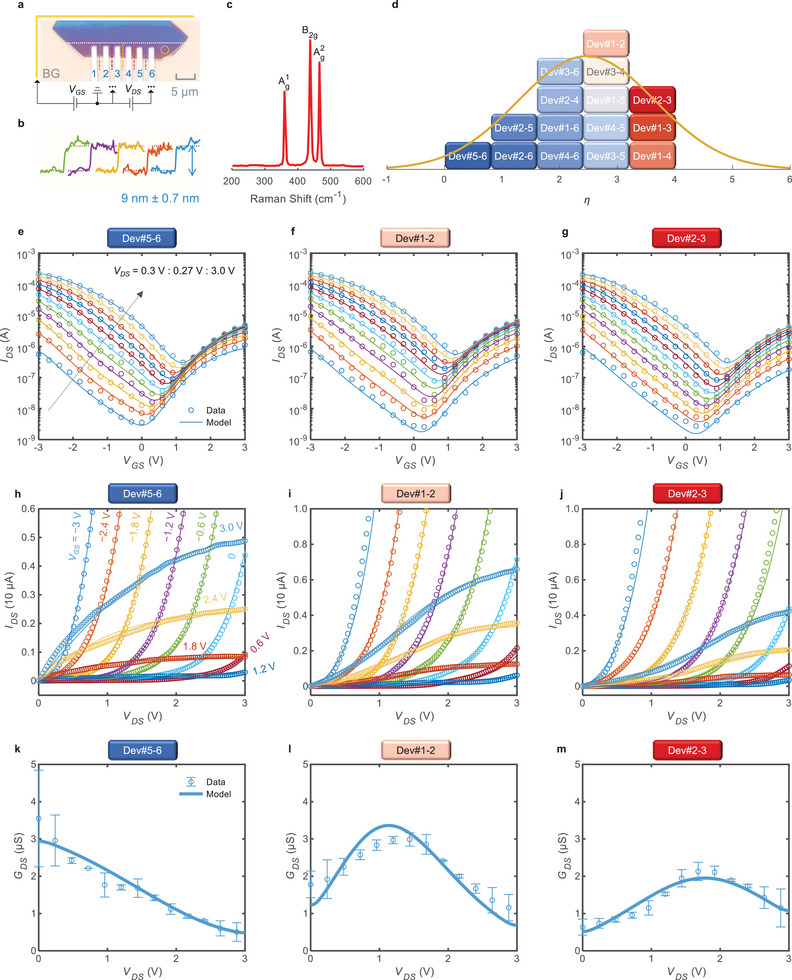
Model verifications with BP‐FETs. a) Optical images of the prepared devices, where the white dashed box highlights the channel region of BP‐FETs; b,c) The AFM and Raman characterizations, respectively. AFM measurements were done for the colored dashed lines in (a). Raman was performed for the region within a yellow circle in (a); d) The distribution of η in the device, where the color of the block represents the value of η; With the classification in (d), three typical‐η devices, Dev#5‐6, Dev#1‐2, and Dev#2‐3, were selected, and e–g) their transfer, h–j) output, and k–m) *V*_GS_ = 3 V drain conductance curves are shown.

BP is a classic 2DM platform that shows ambipolar current transport.^[^ ^46^ ^]^ Here, a batch of BP transistors were experimentally prepared through mechanical exfoliation. An optical image of the devices is shown in **Figure** **2**a, which adopts a back‐gate structure (BG). The BP film of the channel was characterized via atomic force microscopy (AFM) and Raman spectrometry, as shown in Figure 2b,c, respectively, and the thickness was determined to be ≈9 nm (±0.7 nm). Transmission electron microscopy (TEM) combined with energy dispersive spectroscopy (EDS) indicates ≈2 nm natural phosphorus oxide (PO_x_) layers at the interfaces of BP with the Ti and Al_2_O_3_ layers (Note S3, Supporting Information). Hence, the intrinsic BP thickness is around 5−7 nm, less than the thickness characterized by AFM, which is normal for BP.^[^ ^47^ ^]^ Materials obtained by mechanical exfoliation have inherent random dispersion due to uneven external stress and oxidation during tearing and transfer processes.^[^ ^48^ ^]^

We sweep the drain‐source voltage *V*_DS_ from 0 to 3 V and the gate‐source voltage *V*_GS_ from −3 to 3 V to test the BP‐FETs. All combinations of surface electrodes are enumerated. This results in the collection of 15 sets of BP‐FET I‐V data. The selected voltage range covers all possible voltage inputs within the power supply voltage *V*_DD_ = 3 V to make a full comparison, i.e., − *V*_DD_  <  *V*_GS_  <  *V*_DD_ and 0  <  *V*_DS_ <  *V*_DD_. The aim of selecting such broad voltage ranges, of course, is to allow performance evaluation under all practical operation modes of the FET, achieving thorough verification of the model.^[^ ^49^ ^]^

Model simulations and extracted parameters for the measured I–V data are tabulated in Note S4 (Supporting Information). We extract the electrostatic doping profiles^[^ ^5^, ^50^ ^]^ and the doping densities for Dev#2‐3 as examples, which show that the model captures the switching of the dominant carriers in the channel, as given in the Note S5 (Supporting Information). *η* exhibits a bell‐shaped distribution (Figure 2d), which means that devices with nearly ideal Ohmic contact (*η →* 0) and those with significant Schottky contact (*η →* 4) are rare. Most devices are in an intermediate situation. This might be caused by the non‐uniform POx layer as revealed by the TEM graph (Note S3, Supporting Information). It follows that the saturation of the output curves under positive *V*_GS_ get worse as the contact type transitions from Ohmic to Schottky. This can be owed to the increasing voltage drop across the contact junctions, thus leaving less effective electric field dropping through the channel to pinch off the inversion layer. Both Ohmic and Schottky contacts for 2DM FETs have been experimentally reported,^[^ ^5^, ^13^, ^23^, ^24^, ^25^, ^26^, ^27^, ^28^, ^29^, ^30^, ^31^, ^32^ ^]^ as have systems combining different devices types.^[^ ^6^, ^28^, ^51^, ^52^, ^53^, ^54^ ^]^ Process variation is an inherent problem in practical applications. Hence, it is essential to consider the coexistence of Ohmic and Schottky contacts in modeling.

Analyses for the representative devices Dev#5‐6 (with the lowest *η*), Dev#1‐2 (with intermediate *η*), and Dev#2‐3 (with the highest *η*) highlight the capability of our model for describing mixed contact characteristics. Their transfer curves (Figure 2e–g) show a typical ambipolar feature, i.e., the hole current dominates and decreases upon increasing *V*_GS_ when *V*_GS_ < 0, and then the electron current dominates and increases upon increasing *V*_GS_ when *V*_GS_ > 0. However, their output curves (Figure 2h–j) and the derived drain conductances $G_{\mathrm{DS}}≔\partial I_{\mathrm{DS}}/\partial V_{\mathrm{DS}}$ (for *V*_GS_ =  3 V as presented in Figure 2k–m) show significantly different patterns among the devices, especially when the electron dominates (roughly when *V*_GS_ > 1.2 V). The experimental value for *G*_DS_ is obtained through finite difference approximation (FDA) of the *I*_DS_ data. Due to noise contained in the measured signals, the resulting *G*_DS_ by FDA shows local fluctuations. The device with a smaller *η*, Dev#5‐6, exhibits a decreasing *G*_DS_‐trend (Figure 2k), which indicates the intrinsic saturation of the electron‐dominated drain current for an Ohmic‐contact device. In contrast, *G*_DS_ of Dev#1‐2 and Dev#2‐3 undergo non‐monotonic changes (Figure 2l,m), which is an important feature caused by Schottky contacts.^[^ ^24^, ^26^, ^27^, ^33^ ^]^ A similar Schottky feature can be observed for a unipolar MoS_2_ transistor (Note S6, Supporting Information).^[^ ^55^ ^]^ It follows that the model can well reproduce all of these conductivities within error ranges, lending strong support to the Landauer‐QFLPS approach.

**Figure 3:**
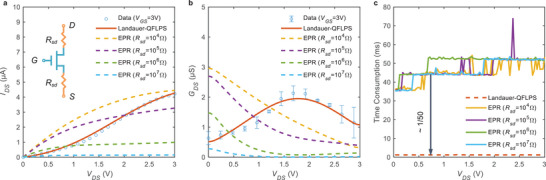
Comparison of the Landauer‐QFLPS and EPR models. a) Output characteristics; b) drain conductance at *V*_GS_ =  3 V; c) Simulation running time. The computer processor used for testing was an Intel Core i7‐10700 CPU (2.90 GHz).

Accurate *G*_DS_ is essential for circuit simulation but is quite difficult to capture due to its derivative nature. As a comparison, the output and drain conductance characteristics of Dev #2–3 with the most pronounced contact effect at *V*_GS_ = 3 V were simulated using the traditional EPR method, as shown in **Figure** **3**. Although the equivalent parasitic resistance *R*_sd_ was scanned over a wide range of values (10 kΩ–10 MΩ), the trend obtained by EPR was still inconsistent with the experimental results, significantly inferior to the Landauer‐QFLPS model. As for the drain conductance, the difference between the two is even more apparent: *G*_DS_ predicted by EPR, which monotonically decreases with *V*_DS_, is entirely wrong, while Landauer‐QFLPS can correctly reproduce the single‐peak of the *G*_DS_ curve rather than a monotonically changed pattern.

The significant improvement in simulation quality can be attributed to the non‐zero BAF introduced. In the EPR model, with such a small resistance, the BAF term is equivalently set to zero, and the current shows a concavely saturated trend due to the increased lateral drain‐source field and the decreased drain electrons as the drain potential increases. In the Landauer‐QFLPS model, a significant contact effect represented by a large BAF strongly suppresses the output curve near *V*_DS_ = 0. When *V*_DS_ increases further, the trend of the corrected model is consistent with that of the intrinsic model.

In addition to accuracy, it should be noted that the model's computing efficiency is significantly superior to the EPR method. This is crucial because the Landauer‐QFLPS model does not explicitly require the solution of Kirchhoff's current law (KCL) equations to characterize the contact effect. Figure 3c shows a comparison of the CPU running time for computation tasks using two different models. The Landauer‐QFLPS model is roughly two orders of magnitude faster than the EPR method.

Furthermore, its capability of covering short‐channel‐length (sub‐20 nm) transistors, including 20 nm BP‐FET,^[^ ^56^ ^]^ 10 nm MoS_2_‐FET,^[^ ^57^ ^]^ 10 nm InSe‐FET,^[^ ^33^ ^]^ and 6.5 nm WSe_2_‐FET,^[^ ^58^ ^]^ are studied as well (Note S7, Supporting Information). The results show good match between the experimental data and the simulations.

## 4 Circuit‐Level Verification

**Figure 4:**
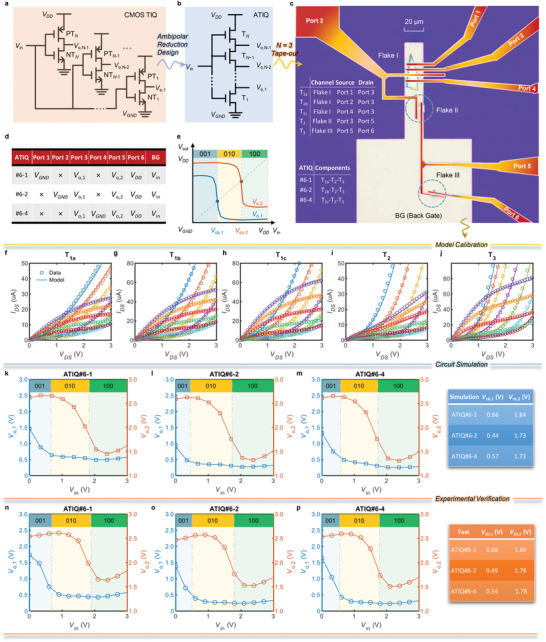
Circuit chip design verification: ATIQ circuit. a) Structure of the threshold inverter quantizer (TIQ) circuit structure based on a conventional CMOS process; b) ATIQ circuit structure based on an ambipolar‐BP process; c) Optical microscope image of 3‐bit ATIQ chip die, where the surface contact electrode is colored with a red‐yellow gradient to visually distinguish it from the back gate (pale yellow), and the light green color is the thin BP layer; d) Circuit test signal code table; e) Ideal electrical characteristic curve of the circuit; f–j) are the model calibration results of the on‐chip BP transistor's device output characteristics; k–m) are the predicted circuit operating characteristics based on the calibrated model, and the table on the right summarizes the predicted boundary code element voltages; and n–p) the corresponding actual test results are shown in the table on the right.

It is essential to examine the circuit‐level simulation capability of the model, in the context of ultimate chip design. Here, an ambipolar TIQ (ATIQ), designed theoretically in our previous work,^[^ ^41^ ^]^ is selected for benchmarking. ATIQ can be used as a quantization element in a flash analog‐to‐digital converter (ADC), which has a compact structure and enjoys low power consumption, thanks to its full utilization of the ambipolar characteristics in 2DMs.^[^ ^41^ ^]^ Note that a conventional TIQ uses parallel CMOS inverters with different flip thresholds to generate quantized levels,^[^ ^59^, ^60^ ^]^ as illustrated in **Figure** **4**a. However, ATIQ stacks ambipolar BP‐FETs in series, as shown in Figure 4b, so that the transistors are multiplexed during operation due to their ambipolarities, saving half of the devices compared with CMOS‐based TIQ. Flash ADC, as the fastest ADC architecture, is usually encumbered by its power consumption and chip‐area costs. The proposed ATIQ unit strikes a balance between high speed, compact size, and low power consumption, thus being a suitable circuit example with practical significance for model verification. Although our work demonstrates the circuit in terms of BP, other 2D materials with ambipolar transport characteristics, such as WSe_2_ and MoTe_2_, are applicable as well.

Several 3‐bit ATIQ instances were fabricated with BP‐FETs as shown in Figure 4c (process details in the Experimental Section). There are three types of configurations for the chip, named ATIQ#6‐1, ATIQ#6‐2, and ATIQ#6‐4, respectively. Each represents a 3‐bit ATIQ instance. Their test schemes are shown in Figure 4d. The ideal voltage transfer curves for a 3‐bit ATIQ circuit are shown in Figure 4e. Due to the different equivalent networks seen by the two ports, two different code boundary voltages *V*_cb,1_ and *V*_cb,2_ are successively generated, thereby realizing a three‐bit one‐hot‐coded quantization. Undoubtedly, if the large‐area thin film preparation method is adopted, the quantization level that can be verified will be higher.

The benchmarking was carried out as follows. First, the I–V data of the BP‐FETs on the chip were measured to calibrate the model. The bias conditions were consistent with the results presented in the previous section. A comparison between model simulations and experimental data is shown in Figure 4f–j (parameters are given in Note S8, Supporting Information). Again, the model accurately reproduces (with an average relative error ≈4%) the experimental results for both T_1a/b/c_ and T_3_, which show negligible contact effects, and for T_2_, which shows significant contact effects.

Next, the three 3‐bit ATIQ circuits were simulated based on the calibrated models. The input analog signal *V*_in_ was applied with the local back gate BG, the power supply voltage *V*_DD_ was connected to Port 6, and the output voltages *V*_o,1_and *V*_o,2_ were measured from Port 3 and Port 5, respectively. To test ATIQ#6‐1/2/4, one should connect *V*_GND_ to Port 1/2/4 accordingly, with the remaining two ports floated. The circuit simulation results are shown in Figure 4k–m, where the model predicts the expected three‐value quantization in all three circuits. Based on the simulations, key circuit parameters including the code boundary voltages *V*_cb,1_ and *V*_cb,2_ were extracted, as summarized in the table beside Figure 4m.

The experimentally measured data are presented in Figure 4n–p. The experimental data are successfully reproduced by the simulations. The extracted code boundary voltages from the experiment show an error within 50 mV, thus quantitatively verifying the model's circuit simulation capability. Optimal designs for code boundary voltages are demonstrated in Note **S9** (Supporting Information).

## 5 Discussion

In order to estimate the significance of the Landauer‐QFLPS model, we compare the spatial profiles of carrier densities and energy bands without and with considering the contact transport, where the former represents the Ohmic‐contact QFLPS theory, while the latter stands for the Schottky‐contact Landauer‐QFLPS model in this work. Dev#2‐3 in the Section “Transistor‐level verification” is selected as the benchmark sample due to its remarkable Schottky characteristics. Dev#2‐3s output curves under *V*_GS_ = 3 V calculated for two cases (Ohmic and Schottky contacts) are referred to from Figure 3a, where the EPR curve with *R*_sd_ = 10^4^ Ω can be regarded nearly Ohmic. Here, we focus on the underlying physics revealed by the model. The spatial distributions are determined from the continuity equations as done in ref. [41] where the parameters are taken from the parameter tables of Dev#2‐3 given in Note S4 (Supporting Information).

**Figure 5:**
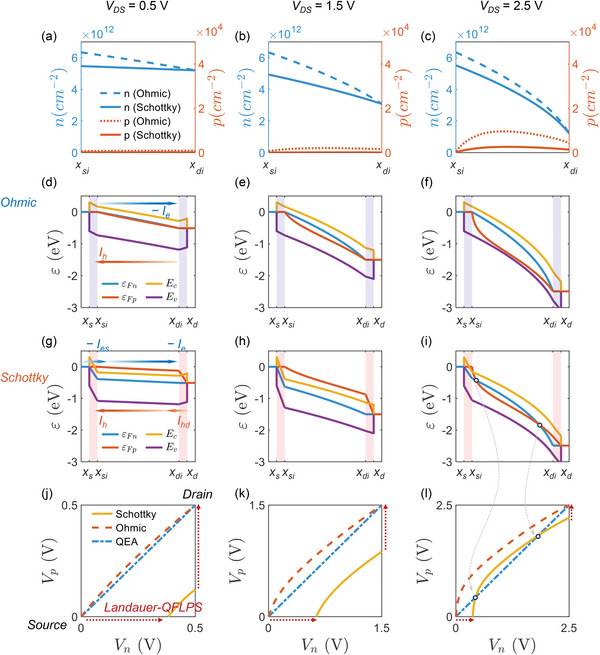
TCAD interpretations of Landauer‐QFLPS. The three columns are obtained under *V*_DS_ = 0.5, 1.5, and 2.5 V, respectively, while *V*_GS_ is kept 3 V. Two types of boundary conditions, i.e., Ohmic and Schottky types, are simulated. a–c) are the carrier density profiles. d–i) are the energy‐band diagrams, where the shaded regions represent the junctions. *ε*_Fn_ and *ε*_Fp_ are identical under Ohmic condition (blue shaded) while distinct under Schottky condition (pink shaded) thus yielding contact transport flows *I*_es_ and *I*_hd_. j–l) are the QFLPS plots (*V*_n(p)_ = −*ε*_Fn(p)_/*q*), where the red dotted arrows indicate the virtual junction‐transport paths that Landauer‐QFLPS model describe.

As shown in the **Figure** **5**, with contact effect enabled in the simulation, the electron injected at the source, and the hole injected at the drain, are both reduced significantly. As drain voltage grows, the electron density is lowered thereby, and the hole density increases, which is much lower than the electrons since a high *V*_GS_ = 3 V bias is applied. At the same time, the junction transport is enhanced upon increasing *V*_DS_. Therefore, a suppressed output curves near the origin is produced.

On the band diagram, the contact‐effect partially inverts the order and endpoints of the electron and hole QFLs, compared with the results in the Ohmic contact simulations, which necessitates the Landauer‐QFLPS formula to include the effect as explained below. For Ohmic contact condition, the electron QFL *ε*_Fn_ is basically higher than the hole QFL *ε*_Fp_ in the channel region and the two QFLs are identical to the external bias at the source/drain boundaries of the channel, which guarantees that the drain current obtained by quasi‐equilibrium approximation (QEA) is accurate and stays closer to the exact solution than that given by the unipolar parallel approximation (UPA) method, since QEA properly considers the electron–hole correlation current as proved by ref. [41]. The order of *ε*_Fn_ > *ε*_Fp_ indicates excess carrier existing in the channel and net recombinations of carriers play a role. However, the enabled Pd/Ti‐BP contacts tend to exhaust the hole in the BP layer (i.e., to set up an anti‐blocking layer for the holes in BP), which requires a net generation to fill the carriers, thus significantly lifting up *ε*_Fp_ so that *ε*_Fp_ becomes higher than *ε*_Fn_. As drain bias grows, holes are accelerated to be injected into the channel from the drain, thus alleviating the lack of holes. Hence, the central part of the channel recovers the order of *ε*_Fn_ > *ε*_Fp_. The change in the QFLs’ spatial profile can be observed on the QFLPS more clearly. The diagonal line of the plane is the QEA path. The Ohmic path that does not consider the contact effect lies over the diagonal line, while the contact‐involved proper paths have much more complicated motions as *V*_DS_ grows. When *V*_DS_ is small, the contact effect pushes the proper Fermi path toward the null‐curl phase region (see ref. [41] for the explanation of the term “null‐curl phase region”), which represents the exhausting effect of carriers by the contact. The modified Fermi path gradually moves toward the full‐curl phase region^[^ ^41^ ^]^ as *V*_DS_ increases and finally intersects with the QEA path. According to the QFLPS theory, the internal Fermi path's configuration in null‐curl phase is less relevant to the resulting drain current, however the endpoints’ deviations would change the results significantly, since the contact transport does not virtually occur on the QFLPS. This gap is exactly what the Landauer‐QFLPS formula does bridge.

## 6 Conclusion

2DM transistors prepared with conventional contact processes generally exhibit a certain proportion of Schottky characteristics, making theoretical modeling difficult. We report a theoretical approach named the Landauer‐QFLPS model, which combines contact currents with channel currents to describe a 2DM‐FET. The unique advantage of the model is its simulation capability for reproducing the non‐monotonic drain conductance observed in the experiments. The universality and practicality of the model are examined in detail to provide a comprehensive assessment, from the underlying physics to top‐level system applications. The Landauer‐QFLPS model should actively promote 2DM transistors as a new electronic device for mature large‐scale system‐level applications.

## 7 Experimental Section

A brief introduction of the QFLPS theory, carrier density model, model parameters, and experimental preparation processes are described below.

### QFLPS Theory

QFLPS is defined as the Cartesian‐product plane of electron and hole QFLs.^[^ ^41^ ^]^ The QFLPS theory features ambipolar integrity and diagram representability compared with the early reported drift‐diffusion formula‐based models.^[^ ^61^, ^62^, ^63^, ^64^, ^65^, ^66^, ^67^ ^]^ Ambipolar integrity means that the Coulomb coupling between electrons and holes in a semiconductor system is effectively included by the quasi‐equilibrium approximation (QEA) derived from the QFLPS theory, which was usually missed in previous works. Simply discarding the terms can yield significant difference compared with the exact continuity equation‐based solutions. Although QEA relies on the strong‐recombination condition, currently reported results indicated that it should hold for mainstream 2D materials. The additional coupling terms given by QFLPS theory improve the physical accuracy yet increase the computational load, too. To overcome this, in this work a zero‐temperature‐limit algorithm with one‐shot convergence was developed in ref. [68] in order that the influence becomes negligible and the theory genuinely stands as a powerful basis for incorporating contact‐effect into the model.

Besides the improvement regarding ambipolarity, QFLPS helps to establish a diagram‐representation of the transistor operation. In the previous work,^[^ ^41^ ^]^ it was proved that QFLPS theory expresses the ambipolar channel current as the Fermi path moving on the QFLPS. By recognizing the dominant carrier type on the phase region, one can read the operation state of the transistor from the QFLPS‐diagram. This method is shown to be even applicable for circuit analysis.^[^ ^41^ ^]^ The principle is that the circuit topology can be converted into the Fermi‐path topology on the QFLPS. In contrast, the diagram analysis method proposed before^[^ ^69^ ^]^ (which works on the plane of *V*_GS_–*V*_DS_ rather than *ε*_Fn_–*ε*_Fp_) mainly focuses on the operation of a single FET device, which restrict the potential applications in the circuit design.

### Carrier Density Model

The functional relations of the electron (*n*) and hole (*p*) densities with their QFLs (ε_Fn_ and ε_Fp_, respectively) are implicitly determined according to the electrostatic–statistic relations (ESRs), which include three equations given as follows.^[^ ^41^, ^68^ ^]^

(i) Gauss's law for the gate‐oxide‐2DM channel system

(10) $$q^{2}\left(n-p\right)/C_{\mathrm{ox}}+\Psi +q\;V_{\mathrm{GS}}=0$$

where Ψ is the electrostatic energy, and *C*_ox_ = ε_r,ox_ε_0_/*t*_ox_  is the specific gate‐oxide capacitance with ε_r,ox_ as the relative permittivity, ε_0_ as the vacuum permittivity, and *t*_ox_ as the gate‐oxide thickness.

(ii) 2D‐electron density

(11) $$n=\Phi_{t}\;D_{e}\ln\left(1+\exp\Phi_{t}^{-1}\left(\varepsilon_{\mathrm{Fn}}-\Psi -\Phi_{n}^{{}^{\prime }}\right)\right)$$

(iii) 2D‐hole density

(12) $$p=\Phi_{t}\;D_{h}\ln\left(1+\exp\Phi_{t}^{-1}\left(\Psi -\Phi_{p}^{{}^{\prime }}-\varepsilon_{\mathrm{Fp}}\right)\right)$$

where Φ_t_ is the effective thermal barrier and $D_{e(h)}=g_{s}g_{v}m_{e(h)}^{*}m_{0}/\pi \hbar^{2}$ is the effective density of states (DOS) for conduction (valence) band electrons (holes) with the spin valley degeneracy set as 1 for simplicity. Here, $m_{e}^{*}=0.15$ and $m_{h}^{*}=0.14$ are used for BP simulations. For MoS_2_, $m_{e}^{*}=0.45$ and $m_{h}^{*}=0.55$ are used. It was reported that the effective mass (EM) approximation adopted here tends to overestimate the resulting drain current compared with ab initio density function theory (DFT) based non‐equilibrium Green's function (NEGF) method.^[^ ^70^ ^]^ This overestimation can be compensated through proper model parameters. A typical example regarding a 5‐nm WS_2_‐FET and its treatment are given in Note S10 (Supporting Information). The shifted Fermi potential barrier for electrons ($\Phi_{n}^{\prime }$) and holes ($\Phi_{p}^{\prime }$) are defined as

(13) $$\Phi_{n}^{{}^{\prime }}=\Phi_{n0}-\Phi_{\mathrm{ms}}-q^{2}N_{f}/C_{\mathrm{ox}}+q^{2}N_{\mathrm{it},e}/C_{\mathrm{ox}}$$

(14) $$\Phi_{p}^{{}^{\prime }}=\Phi_{p0}+\Phi_{\mathrm{ms}}+q^{2}N_{f}/C_{\mathrm{ox}}+q^{2}N_{\mathrm{it},h}/C_{\mathrm{ox}}$$

For BP, the equilibrium electron Fermi potential Φ_n0_ =  0.19 eV, equilibrium hole Fermi potential Φ_p0_ =  0.20 eV, workfunction difference Φ_ms_ =  0.02 eV, and fixed charged impurity *N*_f_ =  5.03 × 10^12^ cm^−2^.

Algorithms to solve the ESRs defined by Equations (10)–(12) and the integrals in Equations (7) and (8) have been reported previously.^[^ ^68^ ^]^

### Model Parameters

The required input parameters include the interface trap densities of electrons and holes (*N*_trp,e_ and *N*_trp,h_), and the thermal activation barrier of the channel carrier (Φ_t_). Other physical parameters, such as the relative effective masses of carriers, $m_{e}^{*}$ and $m_{h}^{*}$, are available in the literature.^[^ ^71^, ^72^, ^73^, ^74^ ^]^ Therefore, a nine‐parameter list {μ_n_, *N*_trp,e_, μ_p_, *N*_trp,h_, Φ_t_, Φ_a,es_, η_es_, Φ_a,hd_, η_hd_} is to be specified. For practical ambipolar devices, electrons and holes can share a set of model parameters that characterize the contact effect (because the ambipolar transport can hardly be observed if the CCL indices of electrons and holes differ too much), and {Φ_a,es_, η_es_, Φ_a,hd_, η_hd_} can be simplified as {Φ_a_,η}, leading to a seven‐parameter list {μ_n_, *N*_trp,e_, μ_p_, *N*_trp,h_, Φ_t_, Φ_a_, η}. For practical reasons, its equivalent list {μ_n_, *N*_trp,e_, μ_p_, *N*_trp,h_, Φ_t_, φ_a_, Φ_a_} with $\varphi_{a}≔\eta \cdot \Phi_{a}$ is trained.

The ideal case points to a situation where all model parameters are constants. However, physical processes in practical devices, such as channel carrier scattering with lattice phonons, carrier–carrier scattering, and Coulomb scattering induced by ionized impurities and defects, render the mobilities no longer constant. Velocity saturation effect (VSE) is another important factor that make mobilities vary with the local electric field.^[^ ^75^, ^76^, ^77^, ^78^, ^79^, ^80^ ^]^ A discussion on the VSE is given in Note S11 (Supporting Information). The local electrical environment should influence the ionization degree of interface traps for electrons and holes. Moreover, the conductivity of the contact ought to be related to the gate‐source voltage, which modulates the barrier height. Based on the above analysis, the model parameters should vary with the external bias, and the rule of variation is rather complicated. However, thanks to careful consideration along the *V*_DS_ variation dimension, a simple but efficient model parameterization strategy is proposed here wherein the model parameters are assumed to only depend on *V*_GS_ and can be approximated using a universal low‐rank function. The coefficients of the low‐rank function serve as parameters to be calibrated by experiments. Linearized Gaussian wavelet (LGW) functions^[^ ^81^ ^]^ are well‐suited for approximating low‐rank functions.

An *N*th (*N* ≥ 2) order LGW with σ‐extension on the bounded interval $B=[V_{\mathrm{GS},\min},V_{\mathrm{GS},\max}]$ is constructed as^[^ ^81^ ^]^

(15) $$H_{N,\sigma }\;\left(V_{\mathrm{GS}}\right)=\int_{-\infty }^{+\infty }\frac{1}{\sqrt{2\pi }\sigma }e^{-{\left(X-V_{\mathrm{GS}}\right)}^{2}/2\sigma^{2}}h{\left\{X_{i},Y_{i}\right\}}_{1\leq i\leq N}\;\left(X\right)dX$$

where *h*{*X*_i_,*Y*_i_}_1 ≤ i ≤ N_(*X*) represents the linear interpolation function of the control points {*X*_i_,*Y*_i_}_1 ≤ i ≤ N_ located at interval B, and the interpolation function value outside that interpolation interval is set to 0. Although the integral form is employed by Equation (15), it can be evaluated analytically with the help of the error function erf(∙)^[^ ^82^ ^]^ as

(16) $$\begin{array}{ccc} H_{N,\sigma }\;\left(V_{\mathrm{GS}}\right) & = & \sum_{y_{i}\in Y\bar{\alpha }}\frac{1}{2}z_{i}\left(v\right)\left[\mathrm{erf}\left(u_{i+1}\right)-\mathrm{erf}\left(u_{i}\right)\right]\\ & & +\;\frac{\xi_{i}\sigma }{\sqrt{2\pi }}\left[\exp\left(-u_{i}^{2}\right)-\exp\left(-u_{i+1}^{2}\right)\right] \end{array}$$

where reduced variables ξ_i_ = (*Y*_i + 1_ − *Y*_i_)/(*X*_i + 1_ − *X*_i_) , *z*_i_ = *Y*_i_  + ξ_i_(*V*_GS_ − *X*_i_), and *u*_i_ = (*X*_i_ − *V*_GS_)/2σ  are defined. LGW is a quasi‐affine transform of the control set {*X*_i_,*Y*_i_}_1 ≤ i ≤ N_.

### Control Points Setting

In principle, both the {*X*_i_}_1 ≤ i ≤ N_ and {*Y*_i_}_1 ≤ i ≤ N_ coordinates can be selected as optimization variables, but this would significantly increase the difficulty of optimization convergence. Therefore, the {*X*_i_}_1 ≤ i ≤ N_ coordinates at interpolation interval B were evenly distributed and only the {*Y*_i_}_1 ≤ i ≤ N_ coordinates were left as optimization variables. To distinguish variables {*Y*_i_}_1 ≤ i ≤ N_ for model parameters α ∈  {μ_n_,*N*_trp,e_, μ_p_,*N*_trp,h_, Φ_t_,φ_a_,Φ_a_}, notation *Y*α is used as presented in parameter tables in Notes S4, S6, S7, S8, and S10 (Supporting Information).

### Rank Optimization

In principle, the higher the value of *N*, the more accurate the approximation. However, due to considerations of convergence cost, only small control sets are used (*N*  =  2,  3).

### Boundary Effect

The odd‐extension technique is employed in the computation here to suppress the well‐known boundary effects.^[^ ^83^ ^]^

### Fabrication Process for BP‐FET

First, a 35 nm Pd/3.5 nm Ti metal stack was deposited as a buried gate electrode on a 300 nm SiO_2_ substrate. Then, using atomic layer deposition (ALD), a 20 nm Al_2_O_3_ dielectric layer was prepared. Next, a mechanical exfoliation process was used to peel thin layers of BP from the bulk material and transfer them to the prepared buried gate substrate using a dry‐transfer method. Next, a 3.5 nm Ti/35 nm drain‐source electrode stack was defined using electron beam lithography. Finally, a 20 nm Al_2_O_3_ passivation layer was encapsulated to protect the BP channel.

### Fabrication Process for BP‐ATIQ

Using an ambipolar‐BP process, the core circuit unit of the 3‐bit ATIQ chip was fabricated in the laboratory. An optical microscope photo of the chip is shown in Figure 4c. A local back‐gate (BG) was fabricated as the input port, and three flakes of the appropriate thickness (Flakes I–III) were transferred to the local gate region. Flake I had a large area, allowing the fabrication of three BP‐FETs, T_1a_, T_1b_, and T_1c_, sharing a common drain electrode (Port 3); the different BP‐FETs could be accessed by choosing the corresponding ports for *V*_GND_ in subsequent circuit tests. The other two smaller flakes, Flake II and Flake III, were used to construct T_2_ and T_3_, respectively, where the drain of T_2_ was connected to the source of T_3_ through Port 5, the source of T_2_ was connected to Port 3, and the drain of T_3_ was led out through Port 6 for use as a power supply *V*_DD_. The high two‐bit devices of the 3‐bit ATIQ are fixed to T_2_ and T_3_. In contrast, the low‐bit device was selected among T_1a_, T_1b_, and T_1c_, denoted ATIQ#6‐1, ATIQ#6‐2, and ATIQ#6‐4, respectively. The mapping relationship among circuits, devices, and ports on the chip is summarized in the embedded table in Figure 4c.^[^ ^55^ ^]^

## Conflict of Interest

The authors declare no conflict of interest.

## Supporting information

Supporting Information

## Data Availability

The data that support the findings of this study are available from the corresponding author upon reasonable request.
