# Supplementary material for: Landauer‐QFLPS Model for Mixed Schottky‐Ohmic Contact Two‐Dimensional Transistors
Source: Adv Sci (Weinh). 2023 Oct 9;10(34):2303734. doi: 10.1002/advs.202303734 (PMC10700253; doi:10.1002/advs.202303734)
Supplement: Supplementary file 1 — Supporting Information [file ADVS-10-2303734-s001.pdf]

ADVANCED SCIENCE
Open Access

Supporting Information

for *Adv. Sci.*, DOI 10.1002/advs.202303734

Landauer-QFLPS Model for Mixed Schottky-Ohmic Contact Two-Dimensional Transistors

Zhao-Yi Yan, Zhan Hou, Kan-Hao Xue, He Tian*, Tian Lu, Junying Xue, Fan Wu, Ruiting Zhao, Minghao Shao, Jianlan Yan, Anzhi Yan, Zhenze Wang, Penghui Shen, Mingyue Zhao, Xiangshui Miao, Zhaoyang Lin, Houfang Liu*, Yi Yang* and Tian-Ling Ren**

Supporting Information

Landauer-QFLPS model for mixed Schottky-Ohmic Contact two-dimensional transistors

Zhao-Yi Yan,[1,2,†] Zhan Hou,[1,2,†] Kan-Hao Xue,[3,4,] He Tian,[1,2,*] Tian Lu,[1,2] Junying Xue,[5] Fan Wu,[1,2] Ruiting Zhao,[1,2] Minghao Shao,[1,2] Jianlan Yan,[1,2] Anzhi Yan,[1,2] Zhenze Wang,[1,2] Penghui Shen,[1,2] Mingyue Zhao,[1,2] Xiangshui Miao,[3,4] Zhaoyang Lin,[5] Houfang Liu,[1,2,*] Yi Yang,[1,2,*] Tian-Ling Ren[1,2,*]*

[1] *School of Integrated Circuits, Tsinghua University, Beijing 100084, China*

[2] *Beijing National Research Center for Information Science and Technology (BNRist), Tsinghua University, Beijing 100084, China*

[3] *School of Integrated Circuits, Huazhong University of Science and Technology, Wuhan 430074, China*

[4] *Hubei Yangtze Memory Laboratories, Wuhan 430205, China*

[5] *Department of Chemistry, Tsinghua University, Beijing 100084, China*

***Corresponding Author**, E-mail: RenTL@tsinghua.edu.cn (T.-L. Ren), yiyang@tsinghua.edu.cn (Y. Yang), tianhe88@tsinghua.edu.cn (H. Tian), houfangliu@tsinghua.edu.cn (H. Liu), xkh@hust.edu.cn (K.-H. Xue)

[†]These authors contributed equally.

Formula derivations, TEM & EDS characterizations for phosphorus oxide, completed I-V data of BP-FETs, MoS_2-FET data simulations, short channel simulations, ATIQ-circuit simulations and optimizations, discussions on effective-mass approximation and velocity saturation effect, are given in this note.

Note 1 | Landauer-QFLPS formula derivation: electrons flow

Based on the Landauer formula, the electron flow injected from the source junction is written as

$$I_{es} = W\frac{q}{\pi\hbar}\int_{-\infty}^{+\infty}\Gamma_{es}(\varepsilon)M_{es}(\varepsilon)[f(\varepsilon,\varepsilon_{Fs}) - f(\varepsilon,\varepsilon_{Fni})]d\varepsilon \#(S1)$$

where ε_{Fni} and ε_{Fs} label the Fermi levels of the intrinsic channel point and the source electrode, respectively. The Fermi-Dirac distribution function is defined as

$$f(\varepsilon,\varepsilon_F) = \frac{1}{1+\exp\left(\frac{\varepsilon-\varepsilon_F}{kT}\right)} \#(S2)$$

where k and T denotes the Boltzmann constant and the temperature, respectively. The modeling for the transmission function $\Gamma_{es}(\varepsilon)$ and the effective density of mode (DOM) function $M_{es}(\varepsilon)$ are given as follows.

A. transmission function $\Gamma_{es}(\varepsilon)$

The transmission rate function $\Gamma_{es}(\varepsilon)$ considers two kinds of transport mechanism: (i) quantum propagation rate that happens on the global energy scale, and (ii) thermal emission rate that occurs only when the energy is higher than the conduction band minimum $\varepsilon > E_{cs}$;

For quantum propagation rate, WKB approximation ($\varepsilon < E_{cs}$) gives that

$$\Gamma_{es}(\varepsilon) = \exp(-2\gamma) \#(S3)$$

where the γ factor is calculated as

$$\gamma(\varepsilon) = \pm\int_0^a \frac{1}{\hbar}\sqrt{|2m_e^* m_0(E_c(x)-\varepsilon)|}\,dx \#(S4)$$

where $E_c(x)$ represents the energy profile function and a denotes the endpoint of the propagation path at the energy level ε so that $E_c(a) = \varepsilon$ and $E_c(0) = E_{cs}$. Energy band profile $\varepsilon_c(x)$ can be determined with Poisson's equation as a parabolic solution as

$$E_c(x) = \varepsilon + \frac{q\rho_s}{\epsilon_s}(x-a)^2 \#(S5)$$

Substituting the expression into $\gamma(\varepsilon)$, one obtains that

$$\gamma(\varepsilon) = \frac{1}{\hbar}\frac{E_{cs}-\varepsilon}{\sqrt{2q\rho_s/m_e^* m_0\epsilon_s}} \#(S6)$$

For $\varepsilon > E_{cs}$, the transmission rate is enhanced by the thermal emission mechanism as

$$\Gamma_{es}(\varepsilon) = \exp\left(-2\gamma + (\varepsilon - E_{cs})/\Phi_{t,es}\right) \#(S7)$$

where $\Phi_{t,es}$ is the thermal emission barrier.

B. density of modes (DOM) function $M_{es}(\varepsilon)$

The DOM function is defined as

$$M_{es}(\varepsilon) = \frac{g_v}{\pi h}\sqrt{2m_{es}^* m_0 K_{es}(\varepsilon)} \#(S8)$$

where g_v is the valley degeneracy, m_{es}^* is the relative effective mass for the electrons in the source metal, and $K_{es}(\varepsilon)$ is the collective kinectic energy of the source electron defined as

$$K_{es}(\varepsilon) = E_{b,es}\exp\left(-\frac{\varepsilon-\varepsilon_{Fs}}{kT_s}\right)\exp\left(\frac{qV_{DS}}{\Phi_{a,es}}\right)\#(S9)$$

where $E_{b,es}$ is the baseline thermal kinectic energy for electron in the source metal, T_s is the effectively local temperature for the source junction, and $\Phi_{a,es}$ is the acceleration barrier for the electrons injected from the source junction.

C. Formulization of I_{es}

Given the definitions for $\Gamma_{es}(\varepsilon)$ (Eq. (S3) and (S7)) and $M_{es}(\varepsilon)$ (Eq. (S8)), one can introduce an index function $A(\varepsilon)$ to simply the calculation so that

$$\Gamma_{es}(\varepsilon)M_{es}(\varepsilon) = e^{A(\varepsilon)}[\mathrm{m}^{-1}]\#(S10)$$

which leads to the explicit expressions for $A(\varepsilon)$ as

$$A(\varepsilon) = \begin{cases} -2\gamma + \dfrac{\varepsilon-E_{cs}}{\Phi_{t,es}} - \dfrac{\varepsilon-\varepsilon_{Fs}}{2kT_s} + \ln M_{es}(\varepsilon_{Fs}) & \varepsilon \geq E_{cs}, \\ -2\gamma - \dfrac{\varepsilon-\varepsilon_{Fs}}{2kT_s} + \ln M_{es}(\varepsilon_{Fs}) & \varepsilon < E_{cs}, \end{cases}\#(S11)$$

With the aid of $A(\varepsilon)$, one can go further to simplify the I_{es} in Eq. (S1) as

$$I_{es} = W\frac{q}{\pi\hbar}\int_{-\infty}^{+\infty} e^{A(\varepsilon)}[f(\varepsilon,\varepsilon_{Fs}) - f(\varepsilon,\varepsilon_{Fni})]d\varepsilon\,\#(S12)$$

Re-write the Fermi window in the square bracket as the integral of the derivative as

$$f(\varepsilon,\varepsilon_{Fs}) - f(\varepsilon,\varepsilon_{Fni}) = \int_{\varepsilon_{Fni}}^{\varepsilon_{Fs}} \frac{\partial}{\partial\varepsilon_F} f(\varepsilon,\varepsilon_F)\,d\varepsilon_F\#(S13)$$

Since the 2D electron density n is proportional to the integral of the f as

$$n(E_c,\varepsilon_F) = \int_{E_c}^{+\infty} D_e f(\varepsilon,\varepsilon_F)\,d\varepsilon\#(S14)$$

where $D_e = g_s g_v m_e^* m_0/\pi\hbar^2$ is the density of states for conduction band electrons in the channel semiconductor with the spin valley degeneracy set as 1, distribution function f can be expressed by the partial derivative of n as

$$f(\varepsilon,\varepsilon_F) = \frac{1}{D_e}\frac{\partial}{\partial\varepsilon_F} n(\varepsilon,\varepsilon_F)\#(S15)$$

and an useful identity holds as

$$\frac{\partial}{\partial\varepsilon_F} f(\varepsilon,\varepsilon_F) = -\frac{\partial}{\partial\varepsilon} f(\varepsilon,\varepsilon_F)\#(S16)$$

Next, substituting Eq. $(S13)$ into Eq. $(S12)$ and interchange the order of integrals, one obtains

$$I_{es} = W\frac{q}{\pi\hbar}\frac{1}{D_e}\int_{\varepsilon_{Fni}}^{\varepsilon_{Fs}}\left\{\int_{-\infty}^{+\infty} e^{A(\varepsilon)}\frac{\partial}{\partial\varepsilon_F} f(\varepsilon,\varepsilon_F)d\varepsilon\right\}d\varepsilon_F\#(S17)$$

Using properties $(S15)$ and $(S16)$, one has

$$I_{es} = W\frac{q}{\pi\hbar}\frac{1}{D_e}\int_{\varepsilon_{Fni}}^{\varepsilon_{Fs}}\left\{\int_{-\infty}^{+\infty} e^{A(\varepsilon)}\frac{\partial^2}{\partial\varepsilon^2} n(\varepsilon,\varepsilon_F)d\varepsilon\right\}d\varepsilon_F\#(S18)$$

Integrating by parts for the inner bracket integral yields

$$I_{es} = W\frac{q}{\pi\hbar}\frac{1}{D_e}\int_{\varepsilon_{Fni}}^{\varepsilon_{Fs}}\left\{\int_{-\infty}^{+\infty} n(\varepsilon,\varepsilon_F)\,[A''(\varepsilon)+A'(\varepsilon)^2]e^{A(\varepsilon)}d\varepsilon\right\}d\varepsilon_F \#(S19)$$

Since a leap of the derivative arises for $A(\varepsilon)$ (c.f. Eq. $(S11)$), $A''(\varepsilon)$ yields a Dirac delta function as

$$A''(\varepsilon) = \frac{1}{\Phi_{t,es}}\delta(\varepsilon - E_{cs})\#(S20)$$

Hence, by neglecting the first-order derivative of $A(\varepsilon)$, one arrives at

$$I_{es} = W\frac{q}{\pi\hbar}\frac{1}{D_e}\int_{\varepsilon_{Fni}}^{\varepsilon_{Fs}} n(E_{cs},\varepsilon_F)\frac{1}{\Phi_{t,es}}e^{A(E_{cs})}\,d\varepsilon_F\#(S21)$$

With the definition of $A(\varepsilon)$ and noting that $E_{cs} - \varepsilon_{Fs} = \Phi_{sb,es}$, one further obtains

$$I_{es} = W\frac{q}{\pi\hbar}\frac{1}{D_e}\frac{1}{\Phi_{t,es}}M_{es}(\varepsilon_{Fs})\exp\left(-\frac{\Phi_{sb,es}}{2kT_s}\right)\int_{\varepsilon_{Fni}}^{\varepsilon_{Fs}} n(E_{cs},\varepsilon_F)\,d\varepsilon_F\#(S22)$$

By gathering the coefficients, it can be written as

$$I_{es} = e^{-\eta_{es}+\frac{qV_{DS}}{\Phi_{a,es}}}\frac{W}{L}\int_{\varepsilon_{Fni}}^{\varepsilon_{Fs}}\mu_n n\,d\varepsilon_F\#(S23)$$

where approximation $n(E_{cs},\varepsilon_F) \approx n(E_c,\varepsilon_F)$ has been made, and the index η_{es} is defined as

$$\eta_{es} = \ln\left[\frac{m_e^* m_0\,\mu_n/q}{L\frac{\sqrt{2m_{es}^* m_0 E_{b,es}}}{8\pi^2\Phi_{t,es}}\exp\left(-\frac{\Phi_{sb,es}}{2kT_s}\right)}\right]\#(S24)$$

Combining with the QEA-model for I_e as

$$I_e = \frac{W}{L}\int_{\varepsilon_{Fd}}^{\varepsilon_{Fni}}\mu_n n\,d\varepsilon_F = \frac{W}{L}\int_{\varepsilon_{Fd}}^{\varepsilon_{Fs}}\mu_n n\,d\varepsilon_F - \int_{\varepsilon_{Fni}}^{\varepsilon_{Fs}}\mu_n n\,d\varepsilon_F\#(S25)$$

one can obtain the final result for I_e by substituting the second integral in Eq. $(S25)$ with Eq. $(S23)$.

Note 2 | Landauer-QFLPS formula derivation: holes flow

Based on the Landau formula, the hole flow injected from drain junction is written as

$$I_{hd} = W\frac{q}{\pi\hbar}\int_{-\infty}^{+\infty}\Gamma_{hd}(\varepsilon)M_{hd}(\varepsilon)\big[f(\varepsilon,\varepsilon_{Fpi}) - f(\varepsilon,\varepsilon_{Fd})\big]d\varepsilon \,\#(S26)$$

where ε_{Fpi} and ε_{Fd} label the Fermi levels of the intrinsic channel point and the drain electrode, respectively. The Fermi-Dirac distribution function is defined as

$$f(\varepsilon,\varepsilon_F) = \frac{1}{1+\exp\left(\frac{\varepsilon-\varepsilon_F}{kT}\right)}\#(S27)$$

where k and T denotes the Boltzmann constant and the temperature, respectively. The modeling for the transmission function $\Gamma_{hd}(\varepsilon)$ and the density of mode (DOM) function $M_{hd}(\varepsilon)$ are given as follows.

A. transmission function $\Gamma_{hd}(\varepsilon)$

The transmission rate function $\Gamma_{hd}(\varepsilon)$ considers two kinds of transport mechanism: (i) quantum propagation rate that happens on the global energy scale, and (ii) thermal emission rate that occurs only when the energy is lower than the valence band maximum $\varepsilon < E_{vd}$;

For quantum propagation rate, WKB approximation ($\varepsilon > E_{vd}$) gives that

$$\Gamma_{hd}(\varepsilon) = \exp(-2\gamma)\,\#(S28)$$

where the γ factor is calculated as

$$\gamma(\varepsilon) = \pm\int_0^a \frac{1}{\hbar}\sqrt{\left|2m_h^* m_0(E_v(x)-\varepsilon)\right|}\,dx\#(S29)$$

where $E_v(x)$ represents the valence band energy profile function and a denotes the endpoint of the propagation path at the energy level ε so that $E_v(a) = \varepsilon$ and $E_v(0) = E_{vd}$. Energy band profile $E_v(x)$ is described with a parabolic function as

$$E_v(x) = \varepsilon - \frac{q\rho_s}{\epsilon_s}(x-a)^2\#(S30)$$

Substituting the expression into $\gamma(\varepsilon)$, one obtains that

$$\gamma(\varepsilon) = \frac{1}{\hbar}\frac{-E_{vd}+\varepsilon}{\sqrt{2q\rho_s/m_h^* m_0\epsilon_s}}\#(S31)$$

For $\varepsilon < E_{vd}$, the transmission rate is enhanced by the thermal emission mechanism as

$$\Gamma_{hd}(\varepsilon) = \exp\big(-2\gamma + (-\varepsilon + E_{vd})/\Phi_{t,hd}\big)\,\#(S32)$$

where $\Phi_{t,hd}$ is the thermal emission barrier.

B. density of modes (DOM) function $M_{hd}(\varepsilon)$

The DOM function is determined by the conservation law as

$$M_{hd}(\varepsilon) = \frac{g_v}{\pi h}\sqrt{2m_{hd}^* m_0 K_{hd}(\varepsilon)}\#(S33)$$

where g_v is the valley degeneracy, m_{hd}^* is the relative effective mass for the electrons in the source metal, and $K_{hd}(\varepsilon)$ is the collective kinectic energy of the source electron defined as

$$K_{hd}(\varepsilon) = E_{b,hd} \exp\left(\frac{\varepsilon - \varepsilon_{Fd}}{kT_d}\right) \exp\left(\frac{qV_{DS}}{\Phi_{a,hd}}\right) \#(S34)$$

where $E_{b,hd}$ is the baseline thermal kinetic energy for hole in the drain metal, T_d is the effectively local temperature for the drain metal, and $\Phi_{a,hd}$ is the acceleration barrier for the holes injected from the drain junction.

C. Formulization of I_{hd}

Given the definitions for $\Gamma_{hd}(\varepsilon)$ and $M_{hd}(\varepsilon)$, one can introduce an index function $A(\varepsilon)$ to simply the calculation so that

$$\Gamma_{hd}(\varepsilon) M_{hd}(\varepsilon) = e^{A(\varepsilon)} \, [\mathrm{m}^{-1}] \#(S35)$$

which leads to the explicit expressions for $A(\varepsilon)$ as

$$A(\varepsilon) = \begin{cases} -2\gamma + \dfrac{-\varepsilon + E_{vd}}{\Phi_{t,hd}} + \dfrac{\varepsilon - \varepsilon_{Fd}}{2kT_d} + \ln M_{hd}(\varepsilon_{Fd}) & -\varepsilon \geq -E_{vd} \\ -2\gamma + \dfrac{\varepsilon - \varepsilon_{Fd}}{2kT_d} + \ln M_{hd}(\varepsilon_{Fd}) & -\varepsilon < -E_{vd} \end{cases} \#(S36)$$

With the aid of $A(\varepsilon)$, one can go further to simplify the I_{hd} in Eq. (S1) as

$$I_{hd} = W \frac{q}{\pi\hbar} \int_{-\infty}^{+\infty} e^{A(\varepsilon)} \left[f(\varepsilon, \varepsilon_{Fpi}) - f(\varepsilon, \varepsilon_{Fd}) \right] d\varepsilon \, \#(S37)$$

Re-write the Fermi window in the square bracket as the integral of the derivative as

$$f(\varepsilon, \varepsilon_{Fpi}) - f(\varepsilon, \varepsilon_{Fd}) = \int_{\varepsilon_{Fd}}^{\varepsilon_{Fpi}} \frac{\partial}{\partial \varepsilon_F} f(\varepsilon, \varepsilon_F) \, d\varepsilon_F \#(S38)$$

Since the 2D hole density p formally is proportional to the integral of the f as

$$p(E_v, \varepsilon_F) = \int_{-\infty}^{E_v} D_h f(-\varepsilon, -\varepsilon_F) \, d\varepsilon \#(S39)$$

where $D_h = g_s g_v m_h^* m_0 / \pi\hbar^2$ is the density of states for valence band holes in the channel semiconductor with the spin valley degeneracy set as 1, distribution function f can be expressed by the partial derivative of p as

$$f(-\varepsilon, -\varepsilon_F) = \frac{1}{D_h} \frac{\partial}{\partial \varepsilon} p(\varepsilon, \varepsilon_F) \#(S40)$$

and an useful identity holds as

$$\frac{\partial}{\partial \varepsilon_F} f(\varepsilon, \varepsilon_F) = -\frac{\partial}{\partial \varepsilon_F} f(-\varepsilon, -\varepsilon_F) = \frac{\partial}{\partial \varepsilon} f(-\varepsilon, -\varepsilon_F) \#(S41)$$

Next, similarly, one obtains

$$I_{hd} = W \frac{q}{\pi\hbar} \int_{\varepsilon_{Fd}}^{\varepsilon_{Fpi}} \left\{ \int_{-\infty}^{+\infty} e^{A(\varepsilon)} \frac{\partial}{\partial \varepsilon_F} f(\varepsilon, \varepsilon_F) d\varepsilon \right\} d\varepsilon_F \#(S42)$$

followed by

$$I_{hd} = W \frac{q}{\pi\hbar} \frac{1}{D_h} \int_{\varepsilon_{Fd}}^{\varepsilon_{Fpi}} \left\{ \int_{-\infty}^{+\infty} e^{A(\varepsilon)} \frac{\partial^2}{\partial \varepsilon^2} p(\varepsilon, \varepsilon_F) d\varepsilon \right\} d\varepsilon_F \#(S43)$$

Integrating by parts for the inner bracket integral yields

$$I_{hd} = W\frac{q}{\pi\hbar}\frac{1}{D_h}\int_{\varepsilon_{Fd}}^{\varepsilon_{Fpi}}\left\{\int_{-\infty}^{+\infty} p(\varepsilon,\varepsilon_F)\,[A''(\varepsilon) + A'(\varepsilon)^2]e^{A(\varepsilon)}d\varepsilon\right\}d\varepsilon_F \#(S44)$$

Since a jump of the derivative arises for $A(\varepsilon)$ in this section, $A''(\varepsilon)$ yields a Dirac delta function as

$$A''(\varepsilon) = \frac{1}{\Phi_{t,hd}}\delta(-\varepsilon + E_{vd}) = \frac{1}{\Phi_{t,hd}}\delta(\varepsilon - E_{vd})\#(S45)$$

Hence, by neglecting the first-order derivative of $A(\varepsilon)$, one arrives at

$$I_{hd} = W\frac{q}{\pi\hbar}\frac{1}{D_h}\int_{\varepsilon_{Fd}}^{\varepsilon_{Fpi}} p(E_{vd},\varepsilon_F)\frac{1}{\Phi_{t,hd}}e^{A(E_{vd})}\,d\varepsilon_F \#(S46)$$

With the definition of $A(\varepsilon)$ and noting that $E_{vd} - \varepsilon_{Fd} = -\Phi_{sb,hd}$, one further obtains

$$I_{hd} = W\frac{q}{\pi\hbar}\frac{1}{D_h}\frac{1}{\Phi_{t,hd}}M_{hd}(\varepsilon_{Fs})\exp\left(-\frac{\Phi_{sb,hd}}{2kT_d}\right)\int_{\varepsilon_{Fd}}^{\varepsilon_{Fpi}} p(E_{vd},\varepsilon_F)\,d\varepsilon_F \#(S47)$$

By gathering the coefficients, it can be written as

$$I_{hd} = e^{-\eta_{hd}+\frac{qV_{DS}}{\Phi_{a,hd}}}\frac{W}{L}\int_{\varepsilon_{Fd}}^{\varepsilon_{Fpi}}\mu_p p\,d\varepsilon_F \#(S48)$$

where

$$\eta_{hd} = \ln\left[\frac{m_h^* m_0\,\mu_p/q}{L\frac{\sqrt{2m_{hd}^* m_0 E_{b,hd}}}{8\pi^2\Phi_{t,hd}}\exp\left(-\frac{\Phi_{sb,hd}}{2kT_d}\right)}\right]\#(S49)$$

Combining with the QEA-model for I_h as

$$I_h = \frac{W}{L}\int_{\varepsilon_{Fpi}}^{\varepsilon_{Fs}}\mu_p p\,d\varepsilon_F = \frac{W}{L}\int_{\varepsilon_{Fd}}^{\varepsilon_{Fs}}\mu_p p\,d\varepsilon_F - \int_{\varepsilon_{Fd}}^{\varepsilon_{Fpi}}\mu_p p\,d\varepsilon_F \#(S50)$$

one can obtain the final result for I_h by substituting the second integral above with Eq. $(S48)$.

Note 3 | TEM and EDS characterizations for the BP-FET devices

TEM analysis is performed for the cross-section of the BP-FET devices used in main text, as indicated in Fig. S1.

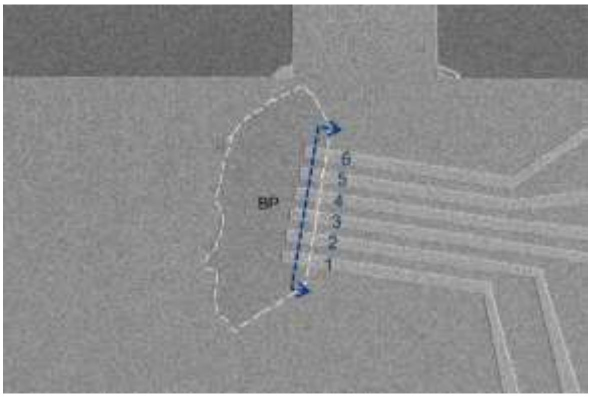

Figure S1 The cut-line (blue dashed line) for TEM analysis

TEM graphs for the defined cross-section are shown in Fig. S2. The gray scale graphs in Fig. S2 exhibit the vertical structures of Pd/Ti/BP/Al_2O_3/Pd stacked layers under six electrodes labelled in Fig. S1, respectively. The 9-nm BP (as measured by AFM in the main text) contains 2-nm around PO_x (varying from 1.3 nm to 2.4 nm as shown in Fig. S2).

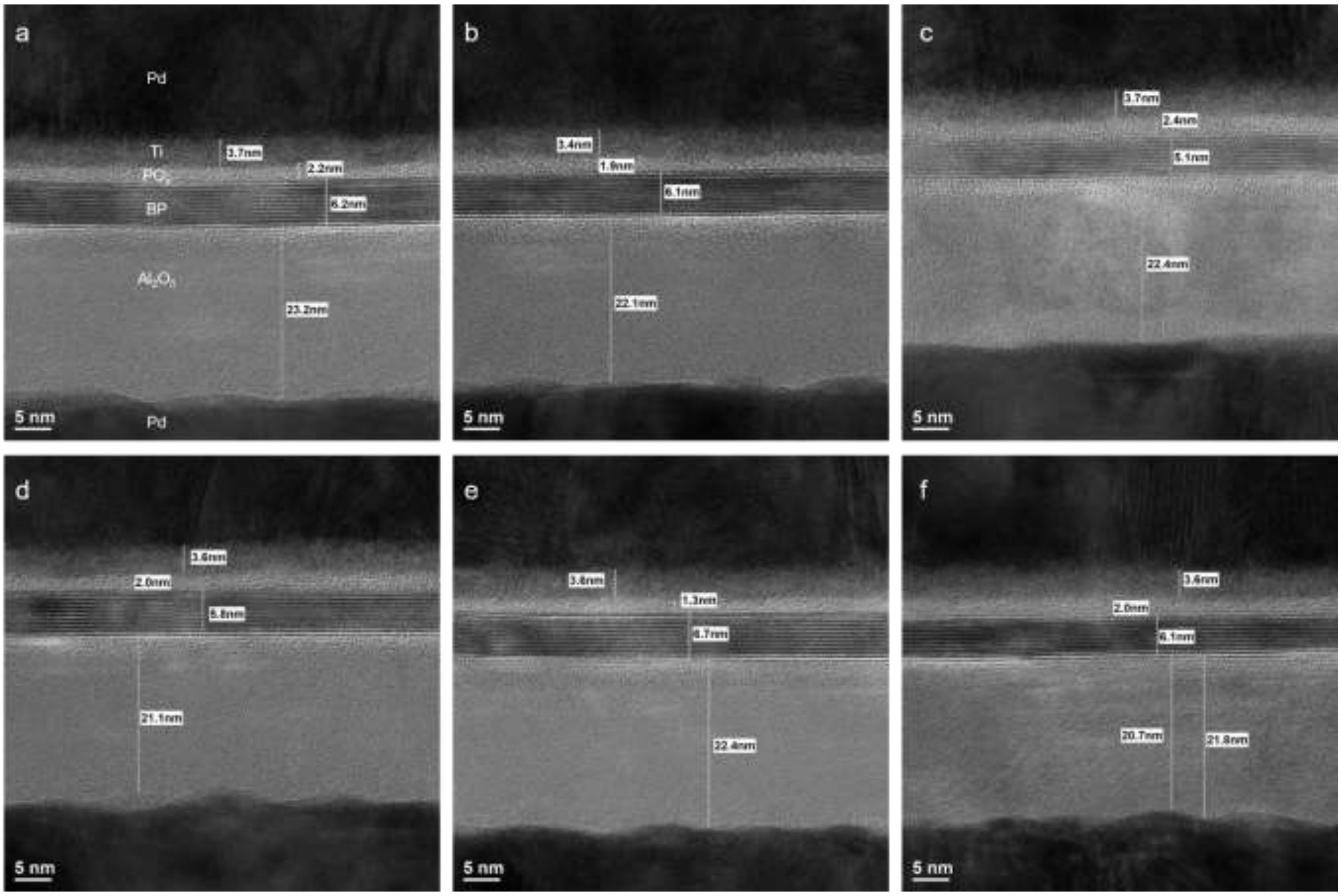

Figure S2 Cross-section view of the BP-FET devices at 5-nm scale. Subplots **a-f** correspond to the electrodes 1-6.

To confirm the element species, we performed the EDS mapping for the stacked layers, as shown in Fig. S3 and Fig. S4. It shows that oxygen exists at the interfaces of BP with Ti- and Pd- layers.

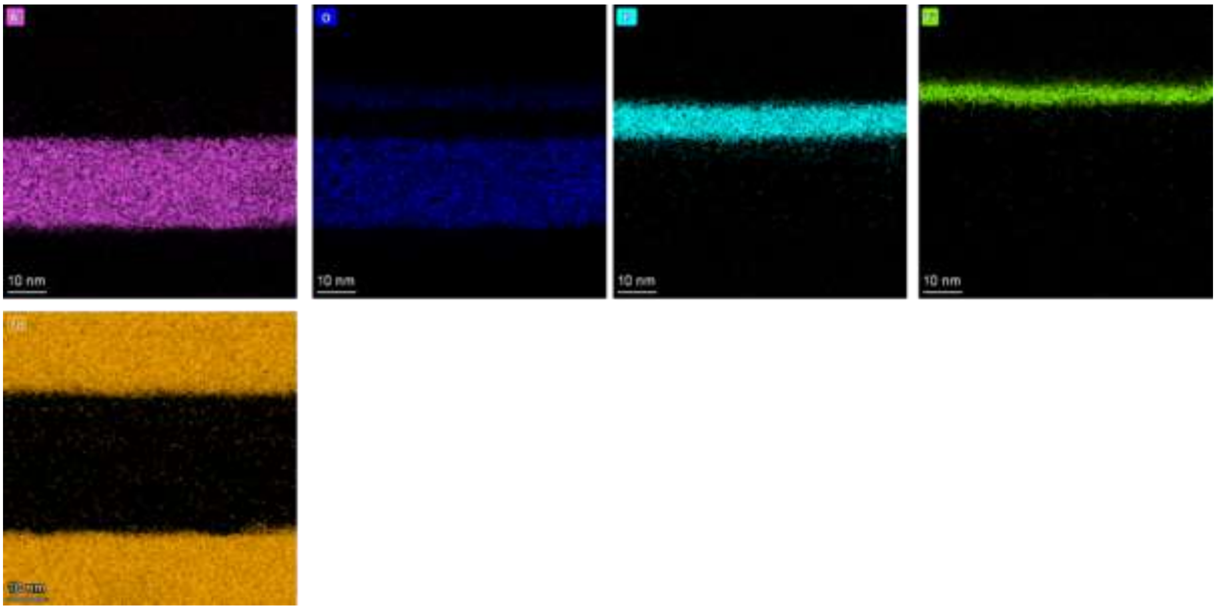

Figure S3 EDS mapping images

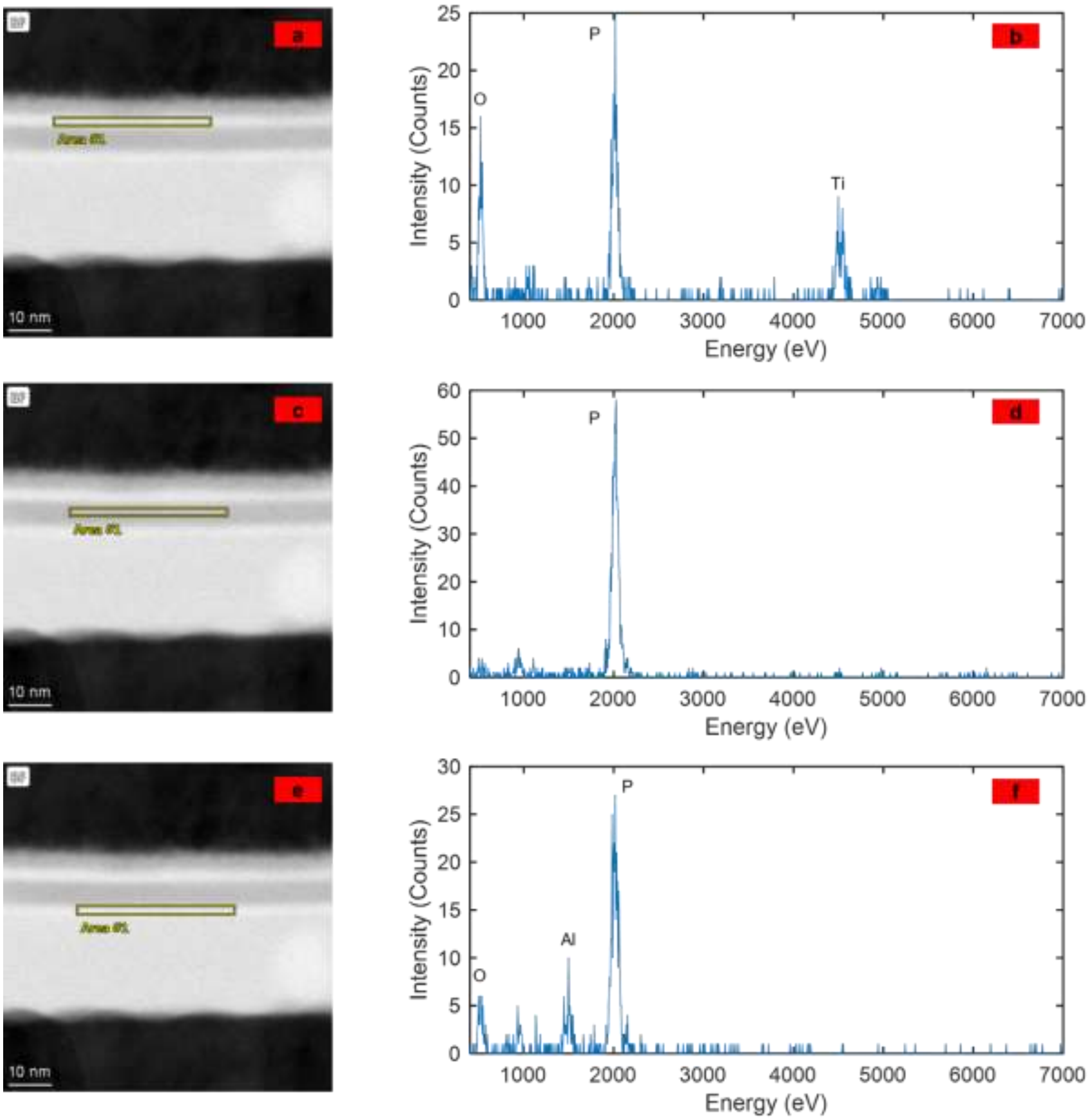

Figure S4 EDS mapping spectrum

Note 4 | BP-FET parameter library and I-V benchmark plots

Table S1 Model parameters library for simulations of the BP-FETs

	$Y\mu_n$ ($cm^2V^{-1}s^{-1}$)	$YN_{it,e}$ (cm^{-2})	$Y\mu_p$ ($cm^2V^{-1}s^{-1}$)	$YN_{it,h}$ (cm^{-2})	$Y\Phi_t$ (eV)	$Y\varphi_a$ (eV)	$Y\Phi_a$ (eV)	σ_s (V)
Dev#1-2								
1	6.00E+00	2.09E+13	1.34E+02	5.81E+12	3.62E-01	1.01E+00	5.68E-01	7.40E-01
2	2.93E+00	7.72E+12	4.00E+02	9.89E+12	5.27E-01	2.09E+00	7.20E-01	*
3	*	*	*	*	3.85E-01	7.00E-01	5.66E-01	*
Dev#2-3								
1	6.01E+00	1.94E+13	8.77E+01	5.81E+12	4.67E-01	1.32E+00	6.14E-01	4.31E-01
2	1.77E+00	6.15E+12	4.01E+02	8.02E+12	5.46E-01	5.30E+00	1.30E+00	*
3	*	*	*	*	2.48E-01	1.56E+00	8.37E-01	*
Dev#3-4								
1	6.00E+00	2.02E+13	1.27E+02	5.71E+12	2.70E-01	-2.03E-01	5.31E-01	6.12E-01
2	2.84E+00	7.48E+12	4.00E+02	9.93E+12	5.09E-01	1.58E+00	5.38E-01	*
3	*	*	*	*	3.61E-01	9.85E-01	4.35E-01	*
Dev#4-5								
1	6.01E+00	1.84E+13	7.63E+01	4.92E+12	2.45E-01	6.26E-01	1.08E+00	1.26E+00
2	1.49E+00	6.22E+12	4.00E+02	7.19E+12	4.74E-01	5.55E+00	2.17E+00	*
3	*	*	*	*	2.42E-01	-2.35E-01	9.61E-01	*
Dev#5-6								
1	9.86E+00	1.64E+13	1.13E+02	5.32E+12	2.64E-01	4.98E-01	5.50E-01	3.07E-01
2	3.66E+00	9.12E+12	5.81E+02	8.02E+12	3.88E-01	-1.00E+00	6.17E+00	*
3	*	*	*	*	3.59E-01	-9.98E-01	3.09E+00	*
Dev#1-3								
1	1.00E+01	2.06E+13	1.25E+02	5.81E+12	4.64E-01	9.93E-01	5.89E-01	4.03E-01
2	3.32E+00	4.53E+12	4.67E+02	9.95E+12	5.72E-01	3.94E+00	1.04E+00	*
3	*	*	*	*	2.34E-01	1.21E+00	5.73E-01	*
Dev#2-4								
1	9.72E+00	2.08E+13	1.43E+02	5.37E+12	3.35E-01	7.16E-01	4.94E-01	6.56E-01
2	3.29E+00	6.86E+12	4.33E+02	7.07E+12	4.73E-01	2.46E+00	1.25E+00	*
3	*	*	*	*	2.92E-01	-6.78E-02	8.00E-01	*
Dev#3-5								
1	6.00E+00	1.98E+13	1.39E+02	5.79E+12	3.25E-01	5.42E-01	5.08E-01	3.05E-01
2	2.80E+00	6.23E+12	4.73E+02	9.02E+12	4.79E-01	1.63E+00	7.70E-01	*
3	*	*	*	*	2.67E-01	5.55E-01	2.74E-01	*
Dev#4-6								
1	6.85E+00	2.01E+13	1.16E+02	5.51E+12	3.39E-01	7.44E-01	4.91E-01	1.76E-01
2	3.22E+00	7.19E+12	6.00E+02	8.90E+12	4.49E-01	2.89E+00	1.92E+00	*
3	*	*	*	*	3.54E-01	-4.00E-01	6.47E-01	*
Dev#1-4								
1	9.09E+00	1.87E+13	1.51E+02	5.81E+12	4.62E-01	8.89E-01	4.69E-01	1.35E+00
2	5.60E+00	5.50E+12	4.39E+02	7.11E+12	5.80E-01	3.88E+00	9.83E-01	*
3	*	*	*	*	1.89E-01	-2.49E-01	8.32E-01	*
Dev#2-5								
1	9.53E+00	2.08E+13	1.30E+02	3.97E+12	2.32E-01	7.51E-01	4.82E-01	4.26E-01
2	3.76E+00	7.55E+12	6.00E+02	8.78E+12	4.38E-01	1.48E+00	1.99E+00	*
3	*	*	*	*	3.82E-01	-1.84E-01	5.66E-01	*
Dev#3-6								
1	6.01E+00	1.81E+13	1.58E+02	5.74E+12	3.68E-01	6.81E-01	4.41E-01	1.37E-01

2	4.30E+00	6.46E+12	6.00E+02	8.84E+12	4.66E-01	2.21E+00	1.16E+00	*
3	*	*	*	*	2.94E-01	4.49E-01	2.56E-01	*
Dev#1-5								
1	9.98E+00	1.76E+13	1.37E+02	3.50E+12	2.47E-01	8.36E-01	4.77E-01	3.43E-01
2	5.25E+00	5.03E+12	6.00E+02	7.89E+12	4.56E-01	2.92E+00	1.34E+00	*
3	*	*	*	*	1.94E-01	-4.18E-01	4.51E-01	*
Dev#2-6								
1	1.00E+01	2.07E+13	1.67E+02	4.52E+12	2.94E-01	7.38E-01	4.03E-01	2.27E-01
2	5.57E+00	8.27E+12	6.00E+02	9.13E+12	4.46E-01	1.30E+00	1.86E+00	*
3	*	*	*	*	4.34E-01	-2.47E-01	6.09E-01	*
Dev#1-6								
1	6.00E+00	1.92E+13	1.50E+02	3.54E+12	3.05E-01	7.37E-01	3.83E-01	2.23E-01
2	5.60E+00	6.09E+12	5.99E+02	8.67E+12	4.60E-01	2.21E+00	1.28E+00	*
3	*	*	*	*	3.71E-01	-3.43E-02	3.22E-01	*

Simulated output/transfer curves in linear/logarithm scale benchmarked with experimental data are shown below

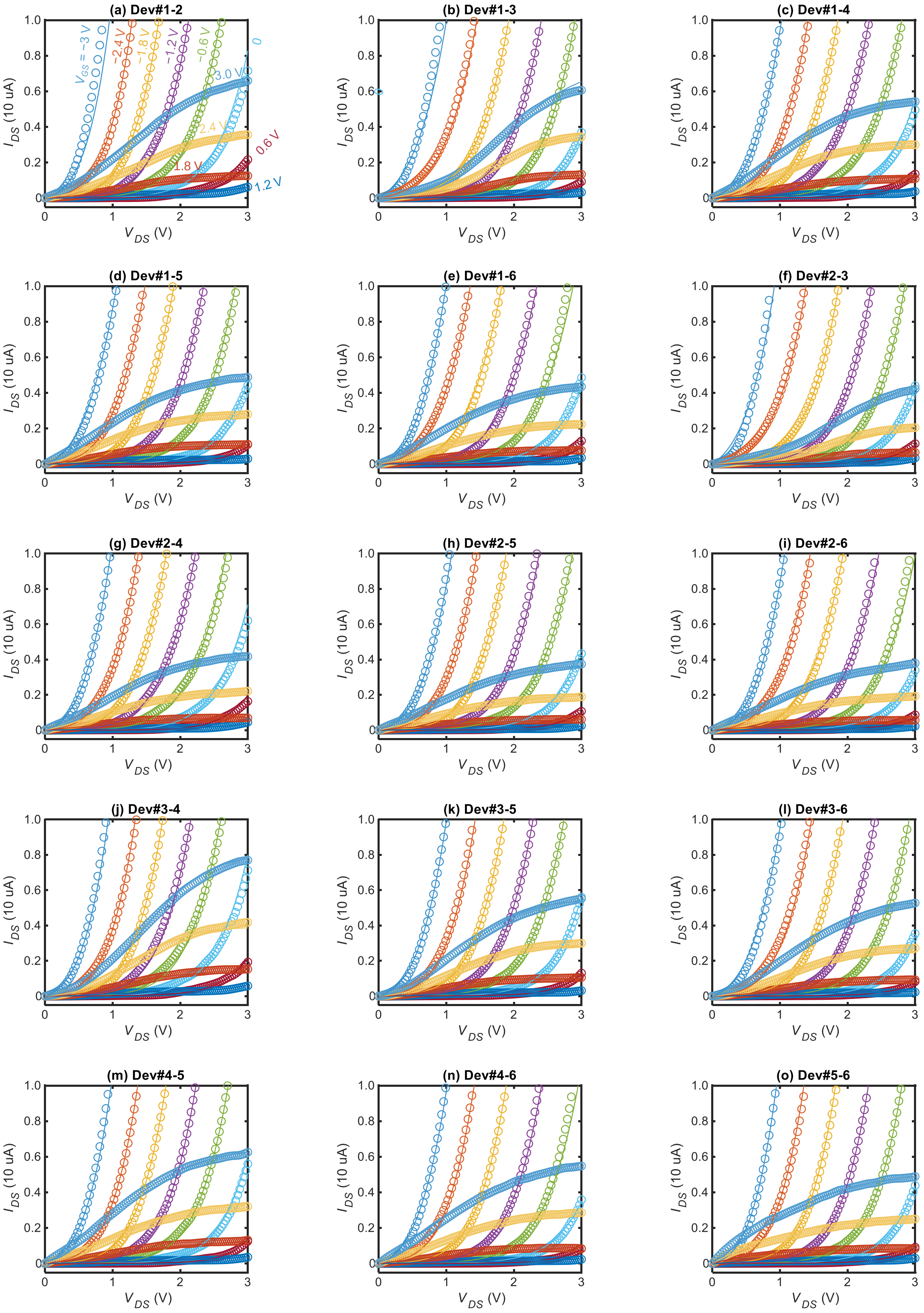

Figure S5 Simulated output curves (lines) benchmarked with experimental data (circles) in linear scale.

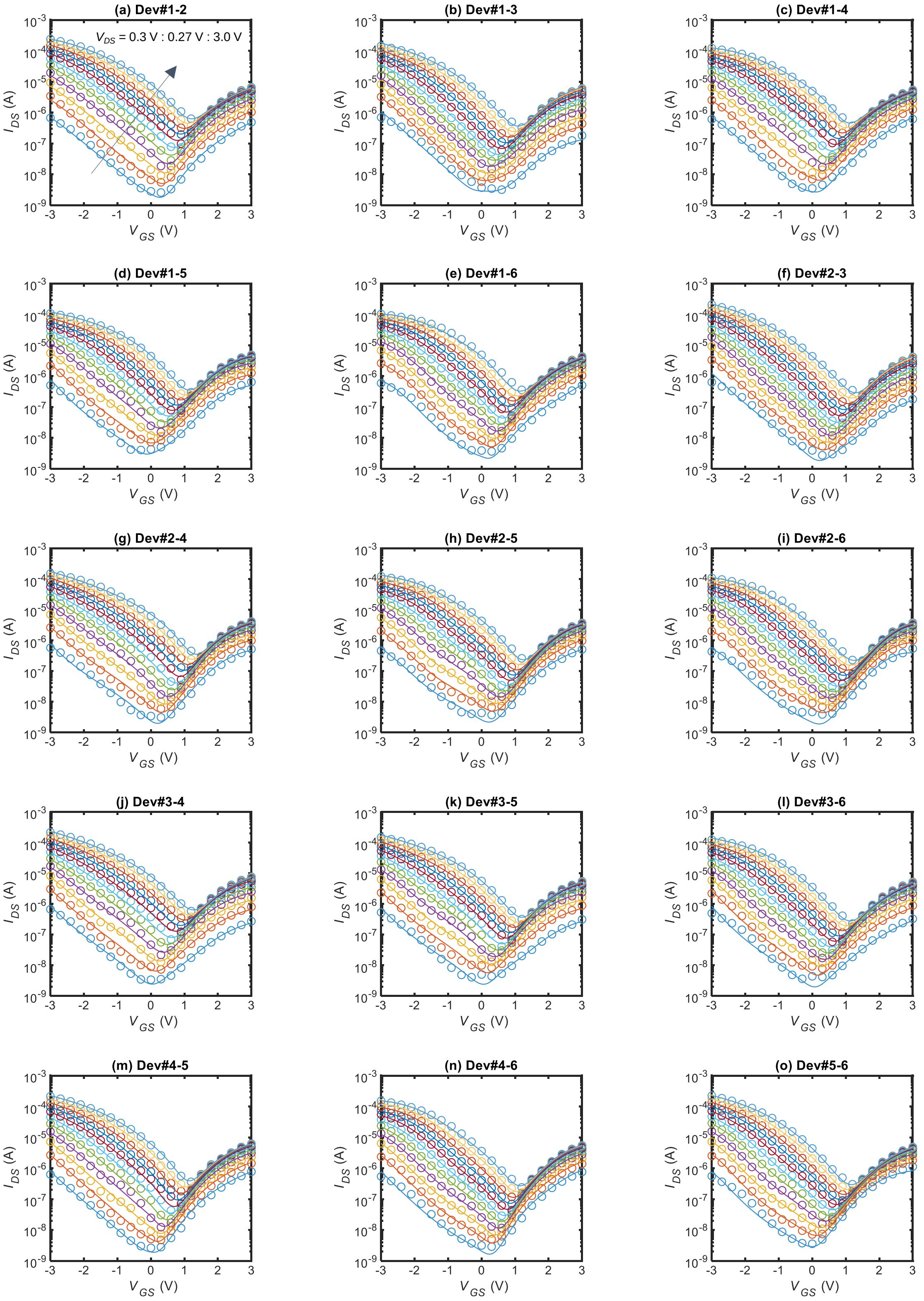

Figure S6 Simulated transfer curves (lines) benchmarked with experimental data (circles) in logarithm scale.

Note 5 | Electrostatic Doping Profiles and Doping Densities

As shown in the Figure S7(a-c), the channel Fermi potential are shifted from E_v neighborhood to E_c nearby, accompanied with the electrostatic doping profile modulated from p$^-$-p$^+$-p$^-$ to n$^-$-n$^+$-n$^-$, thus confirming the dominated carriers changed from holes to electrons.

The source-drain doping densities $N_{dor,sd}$ and $N_{acc,sd}$ are determined by following equations

$$N_{dor,sd} = N_{c,DOS} \ln\left(1 + \exp\left(\left(E_{Fs} - E_{c,s}\right)/\eta kT\right)\right) \#(S51)$$

$$N_{dor,ch} = N_{c,DOS} \ln\left(1 + \exp\left(\left(E_{F,ch} - E_{c,ch}\right)/\eta kT\right)\right) \#(S52)$$

$$N_{acc,sd} = N_{v,DOS} \ln\left(1 + \exp\left(\left(E_{v,s} - E_{Fs}\right)/\eta kT\right)\right) \#(S53)$$

$$N_{acc,ch} = N_{v,DOS} \ln\left(1 + \exp\left(\left(E_{v,ch} - E_{F,ch}\right)/\eta kT\right)\right) \#(S54)$$

where $N_{c(v),DOS}$ represents the equivalent conduction (valence) density of states, E_{Fs} denotes the source Fermi level, $E_{F,ch}$ denotes the channel Fermi level, $E_{c(v),ch}$ represents the conduction (valence) band edge at the channel, and $E_{c(v),s}$ represents the conduction (valence) band edge channel-junction interface. The extracted $N_{dor,ch}$, $N_{acc,ch}$, $N_{dor,sd}$ and $N_{acc,sd}$ are labelled in the Figure S7(d-f), respectively.

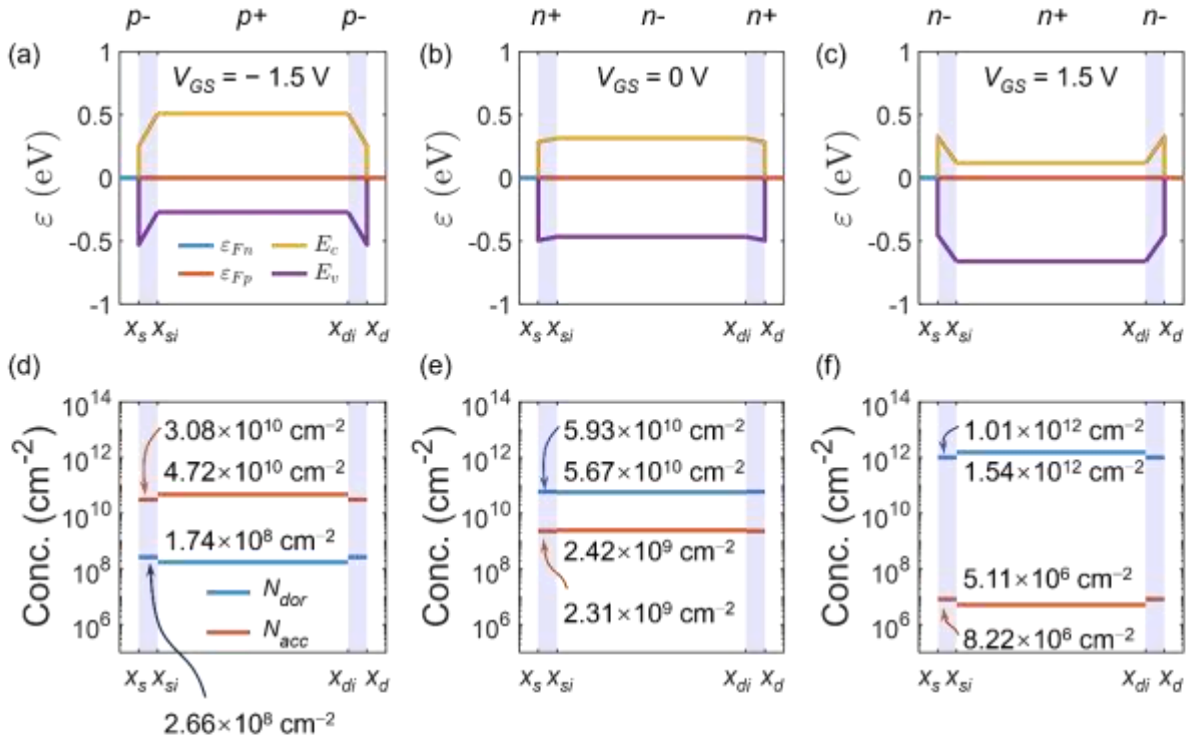

Figure S7 Electrostatic-doping profiles of Dev#2-3. (a-c) are the doping energy diagrams under V_{GS} = −1.5 V, 0, and 1.5 V, respectively, and (d-f) are the corresponding doping densities.

Note 6 | MoS_2 FET Simulation

An MoS_2-FET device is considered below to demonstrate this model for unipolar devices. MoS_2 is a typical n-type 2D layered semiconductor that has attracted much attention due to its suitable mobility and bandgap. Here, MoS_2 transistors[55] were prepared based on back-gate technology for research purposes (Figure S8 (a) and (b)).

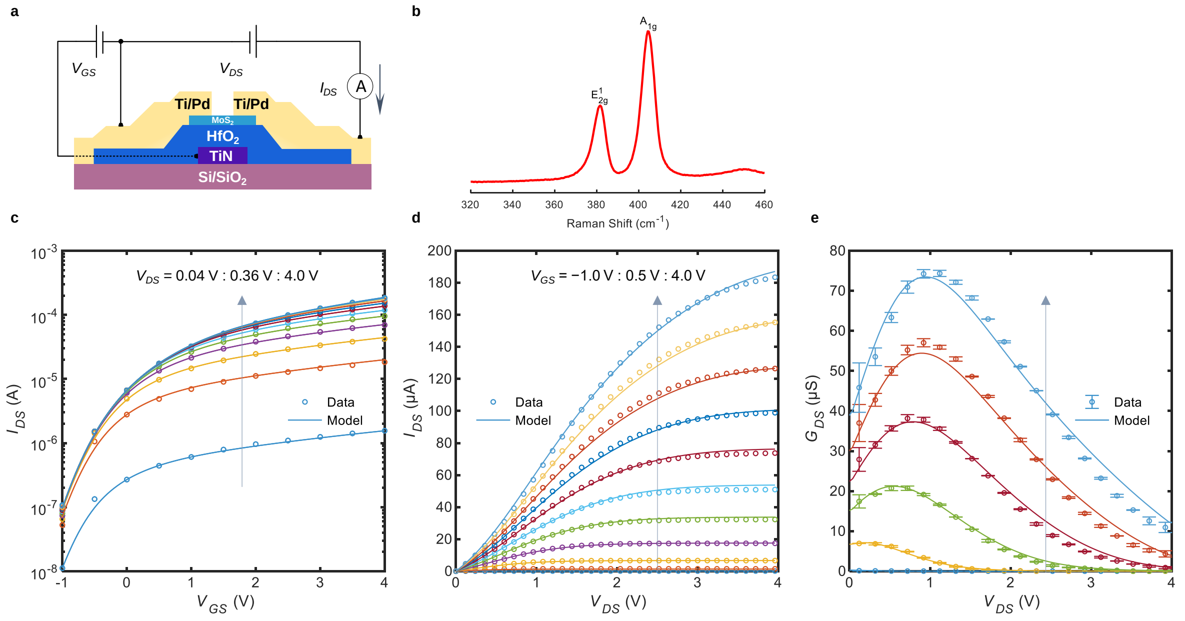

Figure S8 | Verification of MoS_2 devices. **a**, Schematic diagram; **b**, Raman spectrum; (**c**-**e**) Comparison of simulated and measured data for the device's transfer, output, and drain conductance characteristics.

The power supply voltage was 4 V. Unlike BP, the MoS_2 transistor completely turns off at a negative gate-source voltage, so V_{GS} here is only measured until −1V. The experimental data for the transfer, output, and drain conductance characteristics of the prepared MoS_2 transistor are shown in the circular plots in Figure S8(c-e). The output characteristics exhibit a significant contact effect, while the corresponding drain conductance curve shows a clear single-peak feature. The model extraction results (parameters given in Supplementary Note 6) show that, for unipolar devices, the model can still describe the device well and achieve first-order derivative accuracy.

Table S2 Model parameters library for simulations of the MoS2-FETs

T0	$Y\mu_n$ (cm^{2}V^{-1}s^{-1})	$YN_{trp,e}$ (cm^{-2})	$Y\mu_p$ (cm^{2}V^{-1}s^{-1})	$YN_{trp,h}$ (cm^{-2})	$Y\Phi_t$ (eV)	$Y\varphi_a$ (eV)	$Y\Phi_a$ (eV)	σ_s (V)
1	1.86E+00	4.04E+12	*	3.48E+12	2.64E-01	-3.93E-01	2.54E-01	1.00E+00
2	1.86E+00	2.97E+12	*	9.19E+12	4.99E-01	1.93E-01	6.10E-01	*
3	*	*	*	*	2.64E-01	1.40E-01	5.73E-01	*

Fabrication of MoS_2-FET: First, we sputtered a 10 nm TiN electrode onto a 300 nm SiO_2 substrate and left a predefined pattern with a lift-off process. Next, a 15 nm HfO_2 dielectric layer was deposited with ALD as the gate insulator, followed by a spin-coating process to deposit a uniform layer of MoS_2 nanosheet[55]. Next, the sample was heated in an N_2 atmosphere for 1 h at 300 °C. Finally, we used electron beam evaporation to deposit Ti/Pd electrodes.

Note 7 | Short-channel devices simulations

Length-scaling capability is a critical for current EDA developing. Landauer-QFLPS features efficiently solving contact problem. For short-channel transistors, contact problem becomes even more important, since resistances of the intrinsic channel decreases further compared with long-channel cases. To demonstrate our model can be applicable with shorter channel data, we benchmark our model with the other reported experimental results.

Firstly, we collect the transfer and output characteristics of sub-20 nm (include 20 nm) 2D-FETs data achieved in labs, which includes a 20 nm-BP FET reported by Miao et al,[56] a 10 nm-MoS_2 FET by Cao et al,[57] a 10 nm-InSe FET by Jiang et al,[33] and a 6 nm-WSe_2 FET by Xie et al.[58] They stand for the most representatively advanced short-**channel-length** (not merely "gate-length") 2D-transistors reported in the literatures at our best knowledge, thus being appropriate for validating our model. Detailed transistor recipe is summarized in Table. S3. The simulated results are sorted in Figure S9 with model parameters sorted in Table. S4-9.

Table S3 Sub-20 nm 2D-FETs summary

Dataset #	1	2	3	4	5	6
Sweep mode	Transfer Sweeping V_{GS} on [-4 V, 4 V]	Output Sweeping V_{DS} on [-0.1 V, 0 V]	Transfer Sweeping V_{GS} on [-2 V, 2 V]	Output Sweeping V_{DS} on [0 V, 1 V]	Transfer Sweeping V_{GS} on [-0.3 V, 1 V]	Transfer Sweeping V_{GS} on [-10 V, 10 V]
Channel Material	BP	BP	MoS_2	MoS_2	InSe	WSe_2
Channel Length (nm)	20	20	10	10	10	6
Channel thickness (nm)	10	10	0.65 (ML)	0.65 (ML)	2.4	0.7 (ML)
Gate Oxide	10 nm Al_2O_3	10 nm Al_2O_3	6 nm Al_2O_3	6 nm Al_2O_3	2.6 nm HfO_2	10 nm Al_2O_3
Contact metal	Au	Au	Au	Au	Y-InSe	Au
On-off ratio	~10^2	NA	~10^6	NA	~10^6	~10^8
Output-linearity	Ohmic-like	NA	Schottky-like	NA	NA	NA
Polarity	Ambipolar	P-type	N-type	N-type	N-type	Ambipolar

The simulation results demonstrated that our model can universally cover a range of transistors, including both their transfer and output characteristics. As shown in Figure S9(a), the BP-FET reported by Miao et al showed an on-off ratio around 10^2, which makes sense for the 10nm-thick BP flake. It shows nearly ideal Ohmic characteristic for its output curves (Figure S9(b)), which is accurately reflected by our model. The dataset also includes a WSe_2-FET featuring with 6-nm channel length reported by Xie et al (Figure S9(f)), and it shows a ratio up to 10^8, which is larger than that of the BP-FET, indicating our model can capture the wide-range gate-modulation of the carrier density in the channel. The MoS_2-FET reported by Cao et al. shows typical n-type transport of its transfer curve (Figure S9(c)), and Schottky transport behavior of its output curves (Figure S9(d)), which is fundamentally different with normal Ohmic saturation. It is shown that our Landauer-QFLPS model can properly reproduce the convex output curves. Recently, a novel Y-InSe phase doping technique is employed to produce Ohmic contact in InSe-channel transistor with 10 nm channel length.[33] We study its transfer characteristics featuring low DIBL (drain-induced barrier lowering) effect of 22 mV V^{-1} in Figure S9(e), which exhibits well fitness between the experimental data and our model simulations.

We contribute the success in reproducing the electrical characteristics of such short-channel devices to the scalable model structure. This model contains two type of competing transport mechanisms, i.e., the contact tunneling-thermal emission transport and the channel drift-diffusion transport. For short-channel device, the junction tunneling-thermal emission transport would dominant if the channel

scattering can be omitted to mimic the ballistic transport behavior. For the practical cases where the electron-phonon scattering in the channel is important, the drift-diffusion transport would play a role at this time.

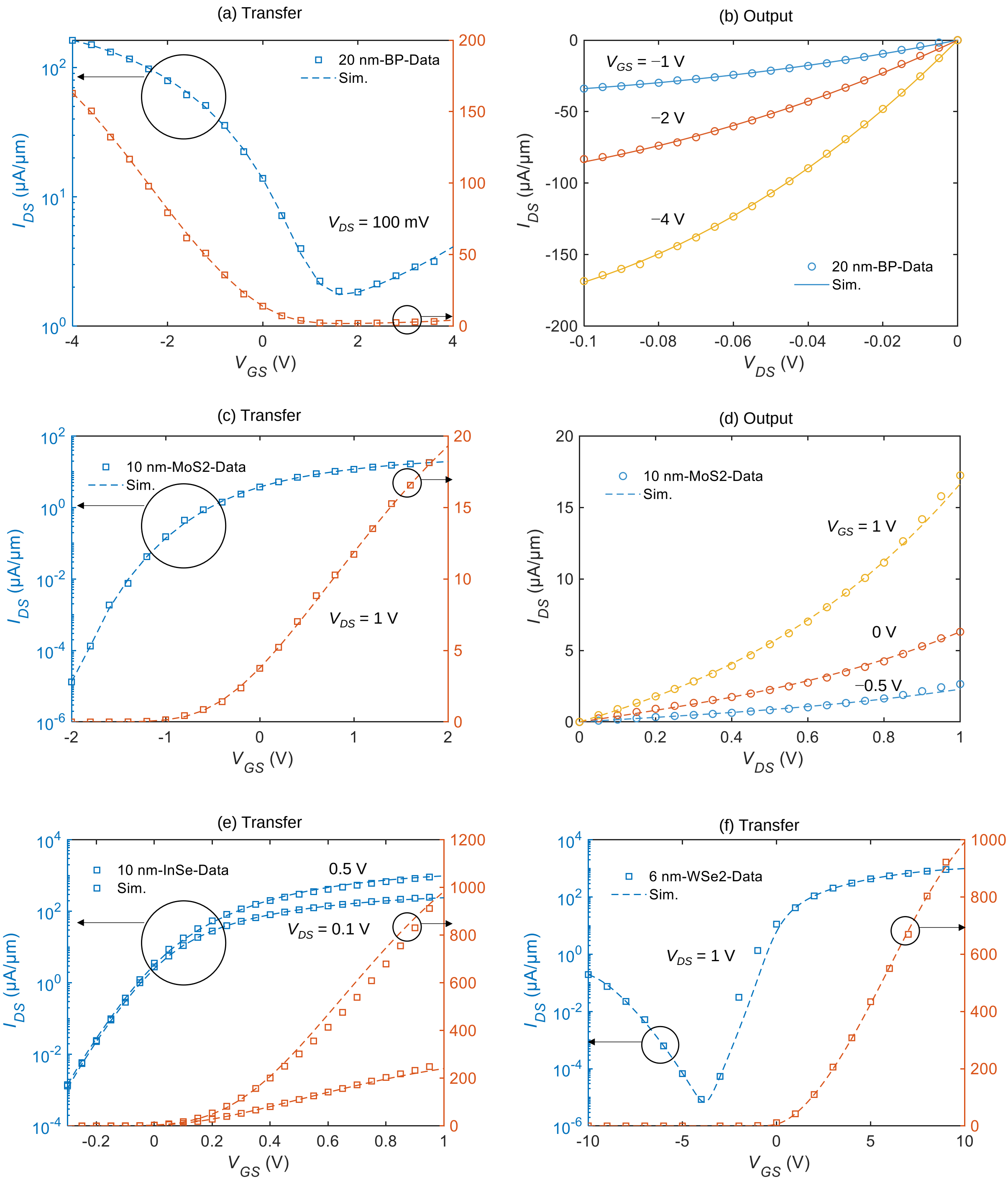

Figure S9 Short-channel transistors' Landauer-QFLPS simulation. (a)-(f) are the transfer and output curves simulations for BP, MoS_2, WSe_2, InSe transistors with channel length down scaling from 20 nm to 6 nm. The experimental data are collected from Refs. [33,56-58], respectively.

Table S4 Model parameters library for simulations of the BP-FET (Transfer)

#	$Y\mu_n$ ($cm^2V^{-1}s^{-1}$)	$YN_{trp,e}$ (cm^{-2})	$Y\mu_p$ ($cm^2V^{-1}s^{-1}$)	$YN_{trp,h}$ (cm^{-2})	$Y\Phi_t$ (eV)	$Y\varphi_a$ (eV)	$Y\Phi_a$ (eV)	σ_s (V)
1	6.59E+00	8.09E+11	2.43E+02	-2.40E+12	1.04E+00	7.68E-01	1.49E-01	3.68E-01
2	1.16E+02	1.40E+13	4.58E+02	7.53E+12	6.49E-01	2.91E-01	5.18E-02	*

3	*	*	*	*	3.52E-01	*	*	*

Table S5 Model parameters library for simulations of the BP-FET (Output)

#	$Y\mu_n$ (cm^{2}V^{-1}s^{-1})	$YN_{trp,e}$ (cm^{-2})	$Y\mu_p$ (cm^{2}V^{-1}s^{-1})	$YN_{trp,h}$ (cm^{-2})	$Y\Phi_t$ (eV)	$Y\varphi_a$ (eV)	$Y\Phi_a$ (eV)	σ_s (V)
1	1.12E+02	3.14E+12	4.71E+02	8.31E+12	7.37E-03	-2.23E-01	1.92E-01	2.89E-01
2	2.99E+02	7.16E+12	1.54E+02	5.82E+12	3.74E-01	-1.97E-02	1.43E-01	*
3	*	*	*	*	4.85E-01	*	*	*

Table S6 Model parameters library for simulations of the MoS_2-FET (Transfer)

#	$Y\mu_n$ (cm^{2}V^{-1}s^{-1})	$YN_{trp,e}$ (cm^{-2})	$Y\mu_p$ (cm^{2}V^{-1}s^{-1})	$YN_{trp,h}$ (cm^{-2})	$Y\Phi_t$ (eV)	$Y\varphi_a$ (eV)	$Y\Phi_a$ (eV)	σ_s (V)
1	2.09E-01	5.78E+12	0.00E+00	1.26E+12	2.23E-01	9.89E-01	1.34E-02	1.00E+00
2	1.08E-01	4.36E+12	0.00E+00	1.26E+12	6.68E-01	9.80E+00	7.40E+00	*
3	*	*	*	*	1.03E+00	*	*	*

Table S7 Model parameters library for simulations of the MoS_2-FET (Output)

#	$Y\mu_n$ (cm^{2}V^{-1}s^{-1})	$YN_{trp,e}$ (cm^{-2})	$Y\mu_p$ (cm^{2}V^{-1}s^{-1})	$YN_{trp,h}$ (cm^{-2})	$Y\Phi_t$ (eV)	$Y\varphi_a$ (eV)	$Y\Phi_a$ (eV)	σ_s (V)
1	1.86E+02	5.84E+11	0.00E+00	1.26E+12	1.68E-01	3.21E+00	7.27E-01	3.62E-01
2	1.62E+02	7.32E+12	0.00E+00	1.26E+12	2.86E-01	1.89E+00	5.44E-01	*
3	*	*	*	*	3.59E-01	*	*	*

Table S8 Model parameters library for simulations of the InSe-FET

#	$Y\mu_n$ (cm^{2}V^{-1}s^{-1})	$YN_{trp,e}$ (cm^{-2})	$Y\mu_p$ (cm^{2}V^{-1}s^{-1})	$YN_{trp,h}$ (cm^{-2})	$Y\Phi_t$ (eV)	$Y\varphi_a$ (eV)	$Y\Phi_a$ (eV)	σ_s (V)
1	1.38E+01	8.19E+12	4.28E+01	2.98E+13	3.82E-01	3.37E+00	2.89E-01	1.00E+00
2	3.41E+00	3.52E+11	1.72E+01	1.19E+13	2.50E-01	2.08E+00	7.64E-01	*
3	*	*	*	*	5.81E-01	*	*	*

Table S9 Model parameters library for simulations of the WSe2-FET

#	$Y\mu_n$ (cm^{2}V^{-1}s^{-1})	$YN_{trp,e}$ (cm^{-2})	$Y\mu_p$ (cm^{2}V^{-1}s^{-1})	$YN_{trp,h}$ (cm^{-2})	$Y\Phi_t$ (eV)	$Y\varphi_a$ (eV)	$Y\Phi_a$ (eV)	σ_s (V)
1	1.38E+01	8.19E+12	4.28E+01	2.98E+13	3.82E-01	3.37E+00	2.89E-01	1.00E+00
2	3.41E+00	3.52E+11	1.72E+01	1.19E+13	2.50E-01	2.08E+00	7.64E-01	*
3	*	*	*	*	5.81E-01	*	*	*

Note 8 | BP-based ATIQ circuits: simulation parameters

Simulation parameters for the BP-FETs used in the ATIQ circuits are sorted as follows.

Table S10 Model parameters library for simulations of the BP-FETs

	$Y\mu_n$ ($cm^2V^{-1}s^{-1}$)	$YN_{trp,e}$ (cm^{-2})	$Y\mu_p$ ($cm^2V^{-1}s^{-1}$)	$YN_{trp,h}$ (cm^{-2})	$Y\Phi_t$ (eV)	$Y\varphi_a$ (eV)	$Y\Phi_a$ (eV)	σ_s (V)
Dev#3-1								
1	5.78E+01	2.54E+12	2.97E+01	5.58E+12	4.53E-01	1.68E-01	4.13E-01	6.69E-01
2	3.31E+01	7.28E+12	5.70E+01	3.83E+12	3.59E-01	-1.91E+00	1.09E-02	*
3	*	*	*	*	4.77E-01	*	5.51E+00	*
Dev#3-2								
1	8.20E+01	2.35E+12	5.90E+01	7.84E+12	4.37E-01	-5.52E+00	1.46E+01	5.37E-01
2	4.91E+01	8.65E+12	1.85E-01	1.73E+12	3.43E-01	4.38E+00	6.53E-01	*
3	*	*	*	*	4.48E-01	*	6.41E+00	*
Dev#3-4								
1	6.49E+01	5.27E+12	4.90E+01	1.07E+13	6.03E-01	1.00E-01	3.39E-01	4.57E-01
2	3.35E+01	8.63E+12	1.50E+00	2.48E+11	3.79E-01	-1.57E+00	2.12E-03	*
3	*	*	*	*	4.68E-01	8.74E-01	6.55E+00	*
Dev#5-3								
1	2.85E+02	2.96E+12	1.51E+02	1.00E+13	4.47E-01	-2.84E+00	2.11E+00	9.91E-01
2	2.97E+02	9.06E+12	4.26E+00	2.35E+12	3.00E-01	3.03E+00	1.58E+00	*
3	*	*	*	*	2.54E-01	5.42E+00	*	*
Dev#6-5								
1	2.97E+02	1.19E+13	1.17E+02	1.56E+13	1.04E+00	1.55E+00	5.14E-01	7.95E-01
2	4.90E+01	9.25E+12	6.00E+02	2.78E+12	4.89E-01	1.87E+00	2.36E+00	*
3	*	*	*	*	3.32E-01	-1.67E-01	*	*

Note 9 | Circuit optimization

The code boundary voltage of the ATIQ#6-1 configuration is higher than the other two configurations. This can be explained by the fact that the lowest transistor T_{1a} of the ATIQ#6-1 has a higher impedance compared to T_{1b} and T_{1c} used in the other two circuits, consistent with the fact that T_{1a} has the longest channel (see Figure 4c in the main text).Optimizing circuit performance is an important function of EDA software. Typically, the ideal code distribution should be uniform. That is, the overall quantization space is evenly divided. Hence, we should have $V_{cb,1}$ =1 V and $V_{cb,2}$ =2 V. However, the initial boundary codes shown above are lower than these ideal values. Although many model parameters can be adjusted to achieve the optimization goal, the most realistic optimization strategy in practical circuit design is still to adjust the channel widths. The following calculations show that this is feasible.

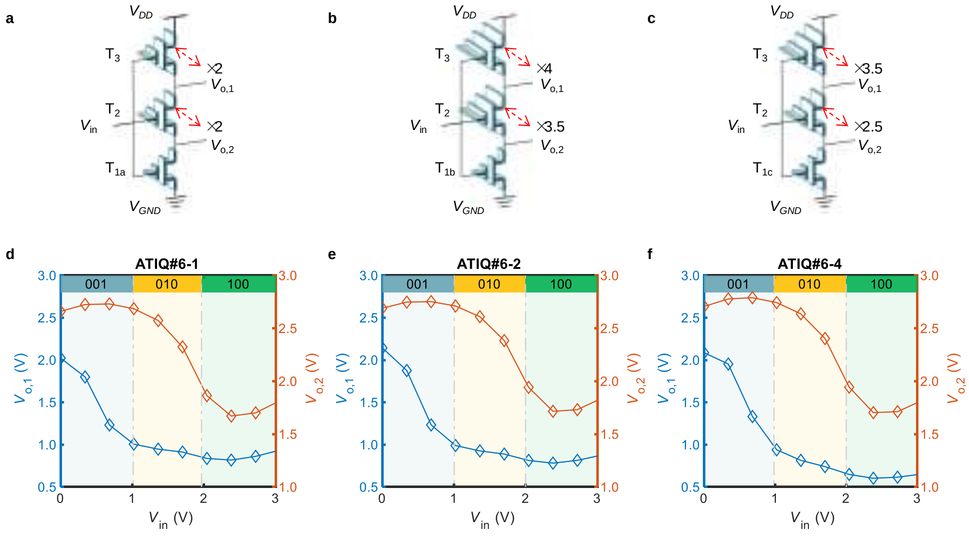

Optimize	$V_{cb,1}$ (V)	$V_{cb,2}$ (V)
ATIQ#6-1	1.01	1.96
ATIQ#6-2	1.00	2.00
ATIQ#6-4	0.98	2.00

Figure S10 Optimizing 3-bit ATIQ circuits based on the Landauer-QFLPS model. a-c are schematics for the width optimizations of the three circuits ATIQ#6-1, ATIQ#6-2, and ATIQ#6-4, respectively. **d-f** are the optimized circuit operating curves. The table on the right summarizes the optimized boundary codes.

Because drain current remains unchanged under overall proportional scaling, we can fix the scaling factor for one device and modify the channel width design of the remaining devices. Because we need to increase the boundary codes, we need to increase the conductivity of the pull-up network, so it can be expected that the correction factors for T_2 and T_3 will be greater than 1. Furthermore, due to the physical half-pitch of the process, the allowed width design is not continuous on the real axis but can only take some discrete values. Obviously, the larger the device size, the more quasi-continuous the values. However, too large a dimension will impair the chip area. Now we consider an extreme case, assuming that the original device width is already twice the half-pitch, so the acceptable width-amplification factor can only be a multiple of 0.5.

Under the constraints, the device widths were redesigned using the calibrated model. The results are shown in Figure S10(a-c), which indicate the magnification ratio of the new widths relative to the original values. With the new designs, a sufficiently uniform code distribution was achieved (Figure S10(d-f)), with fluctuations relative to the ideal distribution not exceeding 40 mV.

Note 10 | Effective-mass approximation

Effective-mass (EM) approximation can overestimate the drain current as strengthened by Ref.[70]. However, the difference between the rigorous *ab initio* DFT-results and the EM results can be compensated by the model parameters, such as mobilities, etc. We simulate a 5-nm WS_2 FET's transfer curve under V_{DS} = 0.6 V proposed in Ref. [70], where both EM-based and DFT-based methods are considered. The results are exhibited in Figure S11, which exhibits that good consistency is obtained between Landauer-QFLPS simulation (labeled as "Sim.") and NEGF methods ("Data"). The extracted electron mobilities in our Landauer-QFLPS model for EM and DFT data under V_{GS} = 0.6 V are around 50 $cm^2V^{-1}s^{-1}$ and 30 $cm^2V^{-1}s^{-1}$, respectively, which reflects the overestimation effect brought by the EM approximation. Other parameters are listed in Table S11.

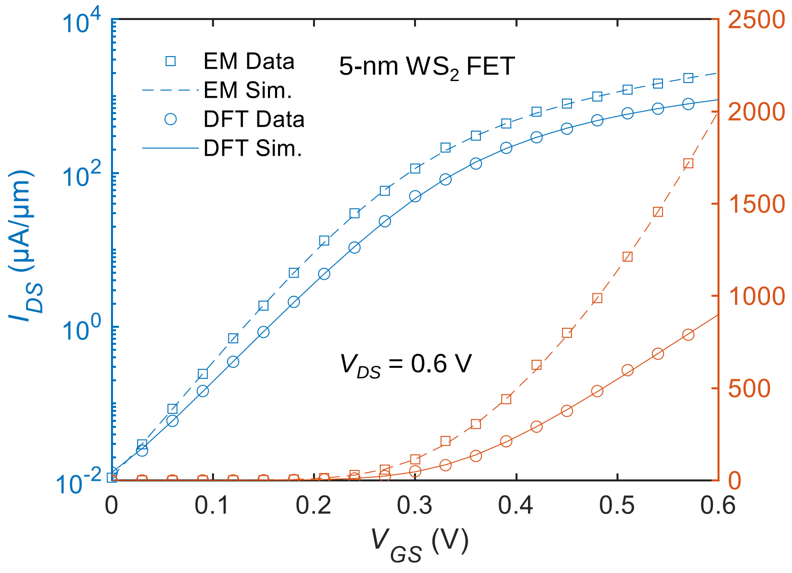

Figure S11 A 5-nm WS_2 FET simulation using Landauer-QFLPS method. The EM and DFT data (squares and circles, respectively) are produced by NEGF method.[70]

Table S11 Model parameters library for simulations of the WS2-FET (EM)

	$Y\mu_n$ ($cm^2V^{-1}s^{-1}$)	$YN_{trp,e}$ (cm^{-2})	$Y\mu_p$ ($cm^2V^{-1}s^{-1}$)	$YN_{trp,h}$ (cm^{-2})	$Y\Phi_t$ (eV)	$Y\varphi_a$ (eV)	$Y\Phi_a$ (eV)	σ_s (V)
EM								
1	2.37E+01	8.24E+12	*	1.26E+12	1.86E-01	7.93E-01	4.16E-01	2.01E-01
2	4.85E+01	4.25E+11	*	1.26E+12	2.49E-01	9.99E+00	2.89E+00	*
3	*	*	*	*	3.87E-01	*	*	*
DFT								
1	3.72E+01	9.18E+12	*	1.26E+12	2.15E-01	1.03E+00	3.88E-01	2.46E-01
2	2.95E+01	5.07E+11	*	1.26E+12	2.57E-01	9.92E+00	2.72E+00	*
3	*	*	*	*	3.37E-01	*	*	*

Note 11 | Velocity saturation effect

Velocity saturation is an important high-order effect for short-channel 2D-FET devices. It typically leads to an earlier saturation point on the output curves, which requires a full V_{GS}, V_{DS}-dependent mobility model. Since we feel it is might be more practical to focus on contact problem as it is the motivation of this work, we choose to include other high-order effect by our V_{GS}-dependent parameter systems. In our model, the earlier saturation point can be captured with V_{GS}-dependent mobility and threshold voltage as validated by the excellent match between the simulation results and the experimental data.

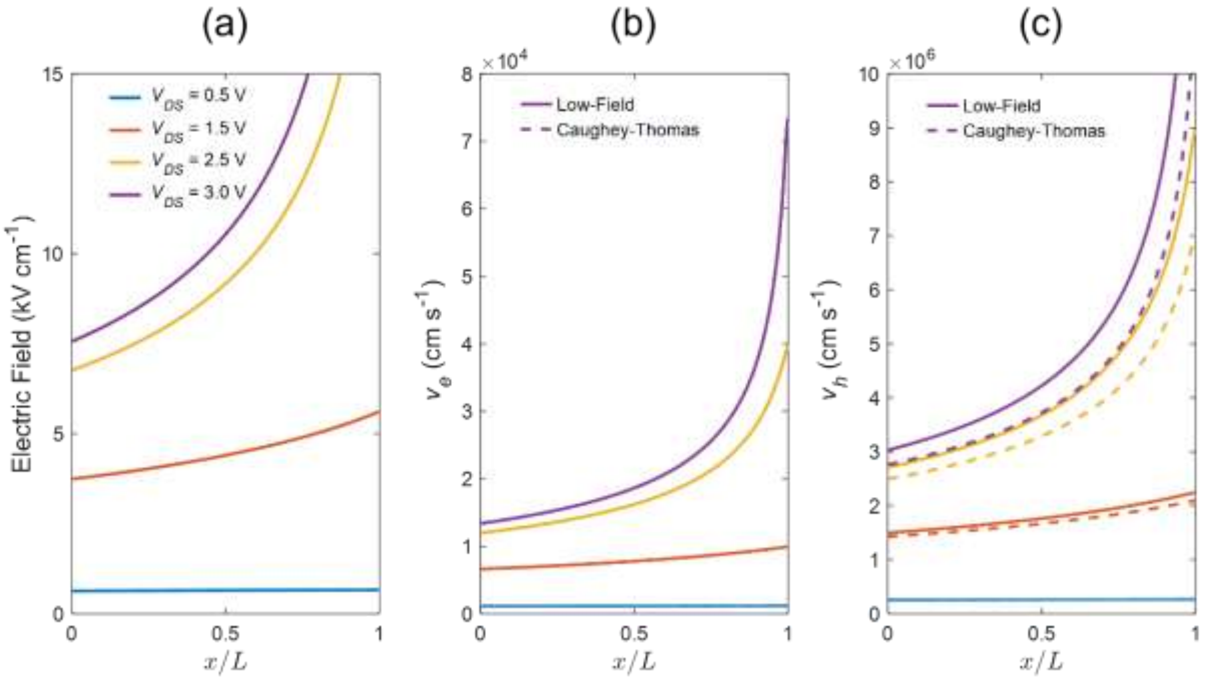

Figure S12 (a) Electric field and (b, c) electron, hole drift velocities under V_{DS} = 0.5 V, 1.5 V, 2.5 V, and 3.0 V, where the solid lines represent the low-field mobility model while the dashed lines represent the Caughey-Thomas V_{DS}-dependent mobility model.

In addition, the velocity-saturation problem in our benchmark devices might be less urgency. We chose BP and MoS_2 devices with micrometer-scale channel-length to validate our model in the manuscript. In such devices, the applied drain voltage V_{DS} ~ 1V can averagely result in around 10 kV/cm electric field in the channel (supported by the TCAD simulation of Dev#2-3 given in Figure S12(a)). And, the extracted mobilities are around 10^2 cm^{2}V^{-1}s^{-1}. Therefore, the average drift velocity under low-field mobility assumption can be determined as 10^6 cm s^{-1}, which is one-magnitude lower than the reported saturation velocity for BP (1.5×10^7 cm s^{-1}), such as in Refs. [76-80]. TCAD simulation shows that significant hole velocity approaching 10^7 cm s^{-1} can be observed at the end of the channel under V_{DS} = 3V, and for most V_{DS} (< 3V) v_h is actually lower than this value through the channel. For comparison, we replace the low-field mobility model currently used in the Landauer-QFLPS model with the classical Caughey-Thomas model, which is written as

$$v_{e(h)}(E) = \frac{\mu_{low,e(h)}E}{\left(1+\left(\frac{\mu_{low,e(h)}E}{v_{0,e(h)}}\right)^{\gamma}\right)^{1/\gamma}}$$

where $\mu_{low,e(h)}$ represents the low-field mobility of electrons (holes), E denotes the electric field, $v_{0,e(h)}$ represents the saturation velocity for electrons (holes) taken as 1.5×10^7 cm s^{-1}, and γ is the empirical

parameters of the Caughey-Thomas model, and taken as 0.8.[76] The results show that a slight suppress can be observed for the holes velocity v_h (Figure S12(c)), while electrons' drift velocity v_e stay nearly unchanged (Figure S12(b)), indicating a slight degree of velocity saturation. Therefore, VSE is less urgent in our examined cases. However, it is worth trying to formally include VSE with the Landauer-QFLPS model in our future work.
